# The Trauma and Mental Health Impacts of Coercive Control: A Systematic Review and Meta-Analysis

**DOI:** 10.1177/15248380231162972

**Published:** 2023-04-13

**Authors:** Susanne Lohmann, Sean Cowlishaw, Luke Ney, Meaghan O’Donnell, Kim Felmingham

**Affiliations:** 1 The University of Melbourne, Melbourne School of Psychological Sceinces, Melbourne, VIC, Australia; 2 Phoenix Australia—Centre for Posttraumatic Mental Health, Department of Psychiatry, The University of Melbourne, Melbourne, VIC, Australia; 3 Queensland University of Technology, School of Psychology & Counselling, Brisbane, Queensland, Australia

**Keywords:** coercive control, intimate partner violence, psychological abuse, PTSD, complex PTSD, depression

## Abstract

Coercive control is an under researched type of intimate partner violence (IPV). The aims of this review were to (a) synthesize all available evidence regarding associations with coercive control and mental health outcomes including post-traumatic stress disorder (PTSD), complex PTSD, and depression; and (b) compare these with associations involving broader categories of psychological IPV. Primary studies which measured associations of coercive control with PTSD, complex PTSD, depression, or other mental health symptoms, were identified via a systematic search of electronic databases (PsycINFO, Medline, CINAHL, Scopus). Eligible studies involved observational designs and reported associations between coercive control and mental health outcomes, among participants who were at least 18 years old. Studies were published in peer-reviewed journals and English language. Random-effects meta-analyses were used to synthesize correlational data from eligible studies. The search identified 68 studies while data from 45 studies could be included in the meta-analyses. These indicated moderate associations involving coercive control and PTSD (*r* = .32; 95% confidence interval [.28, .37]) and depression (*r* = .27; [.22, .31]). These associations were comparable to those involving psychological IPV and PTSD (*r* = .34; [.25, .42]) and depression (*r* = .33; [.26, .40]). Only one study reported on the relationship between coercive control and complex PTSD and meta-analyses could not be performed. This review indicated that coercive control exposure is moderately associated with both PTSD and depression. This highlights that mental health care is needed for those exposed to coercive control, including trauma-informed psychological interventions.

## Background

Intimate partner violence (IPV) is a public health concern that has been linked with long-term mental health consequences including post-traumatic stress disorder (PTSD), depression, anxiety, alcohol and substance use disorders, as well as suicidality (World Health Organization [WHO], 2017). IPV may include physical, sexual, and psychological abuse. Psychological IPV can include verbal aggression and intimidating and belittling behaviors, as well as coercive control (WHO, 2017). Coercive control is a form of abuse where the main goal is to degrade, isolate, and deprive a person of their rights to physical security, dignity, and respect, which puts the victim in a state of terror and entrapment, and includes tactics such as monitoring movements, social isolation, and restriction of access to financial resources, employment, education, or medical care (Pitman, 2016; Stark, 2007, 2013; Stark & Hester, 2019). For instance, abusers may use tactics involving threats to hurt or kill their victims, their children or pets, or isolating them from family, friends, and support services. Coercive control may also involve economic abuse, by threatening economic security and independence (Postmus et al., 2020), intimate partner stalking (Mechanic et al., 2008), as well as reproductive coercion, such as pregnancy coercion or interference with contraception (Miller et al., 2010).

Psychometric measures often differentiate between behaviors attributed to broad forms of all psychological IPV (e.g., belittling, verbal aggression), versus specific dimensions of coercive control (e.g., monitoring, isolation) using subscales. For example, the *Psychological Maltreatment of Women Inventory* (PMWI; Tolman, 1989) provides one of the main measures of psychological IPV which distinguishes coercive controlling behaviors. It includes the emotional/verbal abuse (PMWI-EV) subscale, which captures general forms of psychological IPV, and the dominance/isolation (PMWI-DI) subscale, which captures more targeted features of coercive control.

As coercive control is both a distinct construct and a dimension of broader psychological IPV, it can be unclear whether an abusive behavior occurs in the context of coercive control (Dutton & Goodman, 2005). For instance, verbal threats may or may not reflect coercive control, depending on whether or not these occur in the context of a broader pattern of controlling, isolating, and degrading behaviors. This aligns with Johnson’s (2008) contextual distinction between psychological couple violence which occurs (a) situationally, such as eruptions of heated arguments (potentially involving threats) that are spontaneous and often mutual, and (b) coercive control (referred to by Johnson as intimate terrorism), which is characterized as an enduring pattern of violence, domination, intimidation, isolation, and control. Verbal threats can thus reflect situational couple violence or they can occur within the enduring pattern of domination that characterizes coercive control.

Importantly, coercive control is both highly prevalent, occurring in up to 58% of IPV relationships, and a particularly insidious form of IPV that likely has more severe mental health implications than situational psychological IPV, or even physical IPV that is not embedded in the context of coercive control (Crossman et al., 2016; Kennedy et al., 2018; Stark, 2007). Many studies have shown positive associations between coercive controlling behaviors, including specific forms of economic abuse, stalking, and reproductive coercion, with mental health outcomes including PTSD, depression, and other mental health symptoms. However, the findings are mixed. For example, Beck et al. (2011) have found small associations (*r* = .18), Hines and Douglas (2011) found moderate (*r* = .37), and Taft et al. (2005) found large associations (*r* = .56) for coercive control and PTSD. The mixed results are likely associated with methodological differences such as study settings, measures, and sample sizes.

Systematic reviews and meta-analyses can synthesize the effects of individual studies to overcome the limitations of single studies. To our knowledge, no previous systematic review and meta-analysis has investigated the associations with coercive control and mental health outcomes or compared these effects with the mental health outcomes of general psychological IPV (Pill et al., 2017; Stark & Hester, 2019). Most meta-analyses to date have not focused on psychological IPV and have either solely focused on the mental health implications of physical IPV exposure (e.g., Golding, 1999; Spencer et al., 2019; Stith et al., 2004), or have combined different types of IPV (e.g., Beydoun et al., 2012; Devries et al., 2013; Trevillion et al., 2012). These previous meta-analyses have revealed small to moderate mean correlations between physical or combined IPV and PTSD, depression, anxiety, suicidality, and drug and alcohol use, with the most robust evidence with PTSD and with depression (Devries et al., 2013; Golding, 1999; Spencer et al. 2019; Stith et al., 2004). For instance, Golding (1999) found moderate correlations involving physical IPV with PTSD (*r* = .34) and depression (*r* = .35), while Spencer et al.’s (2019) recent meta-analysis also found moderate correlations with physical IPV and PTSD (*r* = .34) and depression (*r* = .25). Importantly, previous meta-analyses have not distinguished between effects of psychological IPV and coercive control. In part, this may be because the unique impacts of psychological IPV have only been relatively recently more broadly recognized, and because the dimensions of psychological IPV, particularly coercive control, may be more difficult to distinguish and differentiate, when compared to physical IPV (Heise et al., 2019).

Given that meta-analyses have found evidence of the mental health impacts of physical and combined IPV, and the absence of prior systematic reviews of the mental health correlates of coercive control, there is a clear and pressing need for additional systematic examinations of this evidence. This is particularly important as most existing interventions for IPV survivors focus on safety and crisis management, and there is presently a lack of evidence-based psychological programs to support the long-term recovery of those who have been exposed to IPV, particularly coercive control (Hameed et al., 2020). A better understanding of the unique mental health consequences of coercive control would help to inform the development of such evidence-based psychological interventions, and to inform policy and legislation to promote long-term support and recovery (Crossman & Hardesty, 2018).

Moreover, the prolonged and chronic pattern of terror and entrapment of coercive control suggests that such exposures could be uniquely associated with complex PTSD (CPTSD) symptoms (Pill et al., 2017). The latest edition of the *International Statistical Classification of Diseases and Related Health Problems* (11th ed.; [ICD-11], WHO, 2019) includes a diagnostic classification for CPTSD which includes symptoms associated with (a) affective dysregulation, (b) negative self-concept, and (c) disturbances in relationships, which are additional to the diagnostic criteria of PTSD. An essential criterion for an ICD-11 CPTSD diagnosis is the “exposure to an event or series of events of an extremely threatening or horrific nature, most commonly prolonged or repetitive events from which escape is difficult or impossible” (WHO, 2019), which may include prolonged exposure to IPV. Given the chronic terror and entrapment experiences that characterize coercive control, with a typical length of IPV relationships ranging from 15 to 24 months, the likelihood of developing CPTSD in response to coercive control exposure may be high (Kennedy et al., 2018). This may explain in part the more detrimental mental health outcomes compared to other types of IPV (Crossman et al., 2016; Stark, 2007). In fact, it is possible that coercive control may have stronger associations with CPTSD compared to other types of IPV that reflect situational couple violence, because of the prolonged exposure to interpersonal trauma (Cloitre, 2021; Herman, 1992). Therefore, research into the associations between coercive control and CPTSD is important to inform development of effective treatment approaches to deal with the psychological consequences of experiencing coercive control (Karatzias & Cloitre, 2019). However, as far as we are aware, there is no systematic review to-date that has examined the relationship between coercive control and CPTSD.

## Objectives of the Present Study

In this systematic review and meta-analysis we aimed to address limitations of past research by synthesizing the effects of individual studies to provide more precise estimates of the mental health impacts of coercive control on PTSD, CPTSD, and depression. We also add to research by comparing the mental health impact of coercive control with broader dimensions of any psychological IPV. As previous meta-analyses have consistently found small to moderate correlations with physical (or combined types of IPV) and PTSD and depression, and because of the potentially more detrimental mental health impacts of coercive control, when compared to psychological IPV, it was predicted that coercive control would be positively correlated with PTSD, CPTSD, and depression, and that, the strength of these associations, particularly those of CPTSD, would be stronger compared to those of general psychological IPV.

## Method

### Registration and Protocols

The protocol for this systematic review and meta-analysis was preregistered with the *International Prospective Register of Ongoing Systematic Reviews* (PROSPERO) database in June 2021 (registration number: CRD42021252071), while reporting was aligned with guidelines from *Preferred Reported Items for Systematic Reviews and Meta-analyses* (PRISMA; Page et al., 2021).

### Literature Search Strategy

Primary studies examining the associations between coercive control and mental health measures were identified via electronic searches of databases including PsycINFO, Medline, CINAHL, and Scopus. These searches were conducted in May 2021. The search terms for each database are shown in Supplemental Appendix A. All records identified by the search were downloaded into Endnote (Version X9) to remove duplicates. After removing duplicates, the remaining records were uploaded into Covidence Systematic Review Software (2021). Both the title and abstract and the full text screening stages involved two independent reviewers. An exclusion hierarchy was developed by the first reviewer and discussed with the second reviewer before screening. If full text papers could not be obtained, corresponding authors were contacted to obtain full text papers. If authors could not be reached or they did not provide the full text paper, the study was excluded (only two full text papers could not be obtained).

### Inclusion Criteria

Studies were included if they were empirical studies involving observational designs that reported on relationships involving measures of exposure to coercive controlling behaviors and any measure of mental health symptoms or diagnoses (including self-report measures and clinical interviews). Eligible studies had to be written in English language, published in peer reviewed journals, while participants had to be at least 18 years old. There were no exclusions on the basis of gender, ethnicity, regions/country, or publication year. Experimental or intervention studies, and studies that did not report primary quantitative data (e.g., case studies, case series, qualitative studies, reviews, editorials, book chapters) were excluded. Studies were also excluded if the violence was not perpetrated by an intimate partner (e.g., instead perpetrated by another family member), they only reported IPV perpetration, did not differentiate between types of IPV, or did not report any measure of coercive control, or did not report this separately from psychological IPV or other types of IPV. Only studies that measured controlling behaviors were included. Studies that only included measures that do not distinguish dimensions of coercive control, namely the *Conflict Tactics Scale* (CTS; Straus, 1990), Revised Conflict Tactics Scale (CTS2; Straus et al., 1996), the *Severity of Violence Against Women Scale* (SVAWS; Marshall, 1992), and the *Danger Assessment* (Campbell et al., 2009) were excluded. Studies that included measures of coercive control in addition to these scales were included and are listed in the results.

### Quality Assessment

The risk of bias was assessed with the *JBI Critical Appraisal Checklist for Analytical Cross Sectional Studies* (Joanna Briggs Institute, 2017). The quality of all studies was assessed by the first reviewer, while a second reviewer independently assessed 31% of the studies (21 out of 68) which were randomly assigned by selecting every third study in alphabetical order.

### Data Extraction and Coding

The research team developed a coding sheet that included the study design, country, sample size, and gender, recruitment source, sample characteristics, IPV and coercive control measures, mental health measures, statistical methods, and effect sizes. If effect sizes were not reported as either correlations or odds ratios with confidence intervals (CIs), or they could not be computed from the reported data, an email request for the data was sent to the corresponding authors. If the authors did not respond after 1 month or if they were not able to provide the data, the study was excluded from the meta-analysis.

### Data Analyses

Quantitative estimates of associations with measures of coercive control or other forms of IPV with mental health measures were synthesized via a series of random-effects meta-analyses, which account for both within-study and between-study variance and allows for greater generalizability of results (Borenstein et al., 2010). *Comprehensive Meta-Analysis* Version 3 software (Borenstein et al., 2014) was used for these quantitative syntheses. Only cross-sectional studies or longitudinal studies that reported relevant effect sizes at a single time-point (typically study baseline) were included in the meta-analyses. Random effects meta-analyses were performed for associations with coercive control (including economic abuse, stalking, reproductive coercion) with PTSD and depression. Only one study measured CPTSD and meta-analyses for this outcome could not be completed. To examine the strength of associations of coercive control with PTSD and depression, in comparison with the association of psychological IPV with PTSD and depression, additional random effects meta-analyses for the correlations of psychological IPV with PTSD and depression were performed. Only studies that also measured coercive control were included in this comparison, as only these studies met the inclusion criteria for this review.

A Pearson’s *r* correlation coefficient was selected as the effect size index for purposes of reporting and were interpreted based on Cohen’s (1988) guidelines whereby *r* values around 0.10 indicate a small, 0.30 are medium, and values around 0.50 a large effect. Only bivariate effect sizes that could be transformed into a Pearson’s *r* correlation coefficient, such as unadjusted odds ratios and independent group means and standard deviations, were included in the meta-analysis. When a study reported a standardized regression coefficient (β) without reporting a correlation coefficient, the β was imputed as the correlation coefficient (assuming a bivariate model) (Peterson & Brown, 2005). When a study only reported the correlation coefficient for subgroups (e.g., according to gender or ethnicity) without providing a correlation coefficient for the total sample, all subgroup correlation coefficients were transformed using Fisher’s *Z*, and back-transformed after calculating the mean Z value to retrieve the average correlation coefficient (Corey et al., 1998).

Heterogeneity was assessed with the Q and *I*^2^ statistics, where and *I*^2^ value of 25% indicated low, of 50% moderate, and of 75% high heterogeneity (Higgins et al., 2003). A series of exploratory subgroup analyses considering (a) types of coercive control measure (general coercive control measures vs. specific economic abuse, stalking and reproductive coercion measures) and (b) study settings (domestic violence support services/shelters vs. community) were performed to examine potential sources of heterogeneity. Subgroup analyses were only performed when at least six studies were available to be included in a subgroup. Therefore, subgroup analyses comparing gender or countries could not be performed. Statistical significance of subgroup differences was inferred when the 95% CIs for point estimates for each subgroup did not overlap (Cumming & Finch, 2005).

Publication bias was assessed with three tests. First, Duval and Tweedie’s (2000) trim and fill test, which estimates the number of studies missing on the left or right side of the funnel plot, and also estimates the effect size if such hypothetical studies were included. Second, Rosenthal’s (1979) classic fail-safe *N* test, which calculates how many studies with nonsignificant results would be needed to make the mean effect size nonsignificant. A large fail-safe *N* suggests that there is no risk of publication bias. Rosenthal recommends that the minimum fail-safe *N* can be computed by first multiplying the number of effect sizes by 5 and then adding 10 to that number. Finally, Orwin’s (1983) fail-safe *N* identifies the number of potentially missing studies with an effect size of *r* = .00 needed to reduce the mean effect size of each mental health outcome below a small effect size of *r* = .10.

## Results

### Search Results

**Figure 1.**
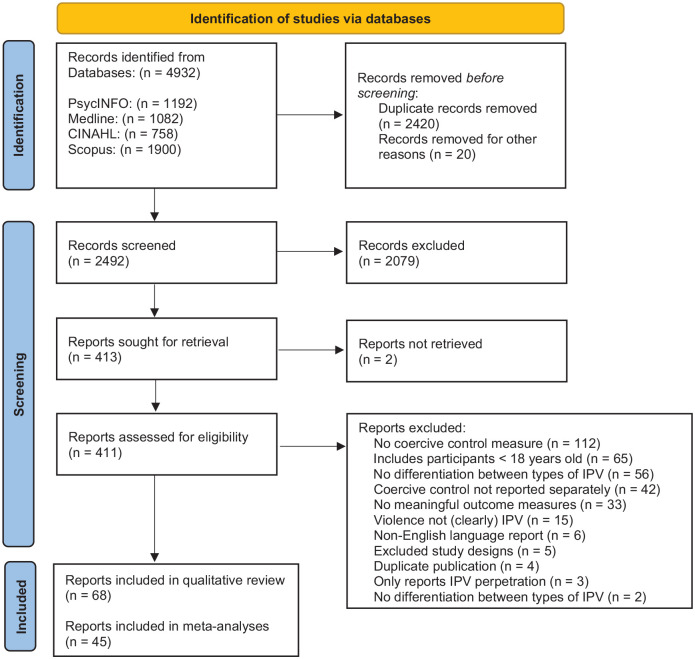
Preferred Reported Items for Systematic Reviews and Meta-analyses flow chart of study selection based on Page et al. (2021).

The combined database search identified a total of 4932 records. After removing 2440 duplicates, there were 2,492 records (PsycINFO = 1,175, Medline = 476, CINAHL = 208, Scopus = 633) imported into Covidence for title and abstract screening. After title and abstract screening, 2,079 records were excluded as ineligible, while 413 studies were passed on to full text screening. After the full text review a further 345 studies were excluded (see Supplemental Appendix B for a list of excluded studies) and 68 eligible studies were remaining. Every title, abstract, and full text record was screened by two independent reviewers. Conflicts were resolved through discussion and consensus. The reviewers identified that the main reasons for conflict were the heterogeneity and overlap of psychological abuse and coercive control measures. Data from 45 studies was available for inclusion in the meta-analyses. The PRISMA flow chart (Figure 1) depicts a summary of the study selection process.

### Description of Studies

The included studies examined associations involving coercive control (including economic abuse, stalking and reproductive coercion) with PTSD, CPTSD, depression, suicidality, anxiety, drug and alcohol use, and transdiagnostic mental health, using a variety of coercive control and mental health measures. Most studies (76%) recruited only women (*k* = 52), while 19% included women and men (*k* = 13), including one study where female and male participants identified as lesbian, gay, bisexual, or transgender. Only 3% of studies included only male participants (*k* = 3), including one study that focused on gay, bisexual, and other men who have sex with men. The vast majority (81%) of studies were conducted in developed countries, with 68% in the United States (*n* = 46). The latter included one study that also included participants from Puerto Rico. These were followed by 6% of studies from Canada (*n* = 4), and 6% from Europe (Denmark: *n* = 2, Sweden: *n* = 2), and Australia (*n* = 1). Other studies were from South America (Brazil: *n* = 2), Africa (South Africa: *n* = 3, Cote d’Ivoire: *n* = 1, Nigeria: *n* = 1, Kenya: *n* = 1, Tanzania: *n* = 1), Asia (Hong Kong: *n* = 1, Malaysia: *n* = 1, South Korea: *n* = 1) and the Middle East (Jordan: *n* = 1). Participants were recruited from a variety of settings, including shelters and domestic violence support services, the community, healthcare settings, and universities. These and other key characteristics included in the qualitative synthesis are summarized in Table 1.

**Table 1.** Description of Studies Included in the Systematic Review in Alphabetical Order.

Study	Country	Study Type	Recruitment	Sample	Age Range (*M; SD*)	Type	IPV Measures			Mental Health Constructs and Measures	
							Coercive control	Psychological	Physical/sexual	Constructs	Measures
* Ahmad et al. (2018)	Malaysia	CS	Com: Post-natal health care facilities	5,727 w	≥18	CC	WHOMCS-CC items	WHOMCS	WHOMCS	Postnatal depression	EPDS (Malaysian)
Alexander et al. (2019)	USA	CS	Com: Community support services	188 w	18–24 (21.3; 2.2)	RC	10-items (Miller et al., 2010)			PTSD, depression	PCPTSD, CESD-SF
* Anderson (2008)	USA	CS 2nd	Com: NVAWS national survey	4,575 w	18–88	CC	NVAWS-CC items			PTSD, depression	IES (adapted), NVAWS 8 depression items
Anderson et al. (2017)	USA	CS	Com: HIV clinic	67 w	18–45	RC	10-items (Miller et al., 2010)			PTSD, depression	PCL-C, CESD
Basile et al. (2004)	USA	CS—2nd	NVAWS: IPV subgroup	380 w	<18 (40)	ST	NVAWS-ST Items	NVAWS verbal abuse + CC	CTS	PTSD	IES (adapted)
*Beck et al. (2011)	USA	CS	IPV survivors—university research clinic	63 w	18–64 (36.75; 11.62)	CC	PMWI-DI	PMWI-EV	Interview devised by authors	PTSD	CAPS for DSM-IV
* Broughton and Ford-Gilboe (2017)	Canada	LO—2nd	IPV—subsample from WHES	157 w	20–64 (40.6; 9.6)	CC	WEB			Depression	CESD
* BubriskiMcKenzie and Jasinski (2013)	USA	CS 2nd	Com: IPV sample CWHRS	705 w	≥18 Bl: (31.07; 9.46) His: (29.08; 7.79	CC	CWHRS Power and Control Scale		CTS2	PTSD, Depression	PSS-I CWHRS-based on MOS
Coker et al. (2002)	USA	CS—2nd	Com: NVAWS national survey	6,790 w 7,122 m	18–65	CC	NVAWS-CC items	NVAWS-verbal abuse	CTS	Depression Drug and Alcohol Use	SF-36 NVAWS Drug and Alcohol items
* Damra and Abujilban (2021)	Jordan	CS	Com: hospital gynecology/obstetric service	300 w	18–49 (32; 6.4)	CC	WHOMCS-CC items	WHOMCS	WHOMCS	Depression	BDI for Jordanian culture
* Dokkedahl et al. (2021)	Denmark	CS	Shelter	147 w	≥18 (34.6; 10.1)	CC	PMWI-DI	PMWI-EV	CTS2	PTSD	ITQ, HTQ, TSC-26
* Dutton and Painter (1993)	Canada	LO	IPV—shelters, support programs	75 w	≥18 (31.4)		PMWI-DI	PMWI-EV	CTS	PTSD	TSC-33
Dutton et al. (1999)	USA	CS	DV support service	149 w	18–58 (30)	CC	PMWI-DI	PMWI-EV	CTS2	PTSD, Depression	PSS-SR, CESD
* Emery et al. (2019)	South Korea	CS	Com	462 w	≥18	CC	NVAWS-CC items			Alcohol Use	CAGE
Fleming et al. (2012)	USA	CS	Com	192 w	≥18 (55.04; 3.92)	ST	NVAWS-ST items	CTS2	CTS2	PTSD	PCL-C
* Gibbs et al. (2018)	South Africa	CS	Com	677 w	18–30 (23.7)	EA	WHOMCS-CC	WHOMCS		Depression Suicidal Ideation	CESD SI: 1-item
Gou et al. (2019)	Canada	LO	Com	98 w 98 m	≥18 w: (29.98; 5.49) m: (32.03; 5.51)	CC	PMWI-DI		CTS2		CESD
Grace et al. (2020)	USA	CS	University students	354 w	≥18 (20.88; 2.31)	CC, RC	2 RC questions CAS	CAS	CAS	Depression Drug and Alcohol Use	CESD-R MFDAQ
* Groves et al. (2012)	South Africa	CS	Com: HIV	1,500 w	≥18 (27.29; 5.36)	CC	SRPS	adapted WHOMCS	adapted WHOMCS	Depression, Anxiety	HSCL-25
Hardesty et al. (2019)	USA	LO	Com: mothers who filed for divorce	135 w	20.83–53.92 (35.22; 7.02)	CC	PMWI-DI		CTS2	PTSD, Depression	PCL-C-SF CESD-SF
Hayes and Kopp (2020)	USA	CS—2nd	Com: National NISVS	7,433 w 6266 m	≥18	CC RC	Number of CC, RC past year	Number of psych. IPV past year	Number of physical IPV past year	Mental Health	Self-rated
* Hazen et al. (2008)	USA	CS	Com: Healthcare service users	282 w	18–45 (27.74; 7.12)	CC	PMWI-DI-SF	PMWI-EV-SF	CTS2	Depression, Anxiety	BSI
* Hedin and Janson (1999)	Sweden	CS	Com: Antenatal clinics	207 w	18–35 (29.5, 4.5)	CC	PMWI-DI	PMWI-EV	SVAWS	PTSD, Anxiety	TSC-33 STAI
* Hines and Douglas (2011)	USA	CS	IPV support services (help-seeking group), Com	822 m (302 IPV 520 com)	18–59 IT: (40.49; 8.97) Com: (43.68; 10.88)	CC	PMWI-DI	CTS2	CTS2	PTSD	PCL-C
* Hines and Douglas (2012)	USA	CS	IPV services (help-seeking group), Com	822 m (302 IPV 520 Com)	18–59 help-seek. (40.49; 8.97) Com: (43.68; 10.88)	CC	PMWI-DI	CTS2	CTS2	Drug and Alcohol Use	NWS Alcohol and Drug Use scale
* Hines and Douglas (2018)	USA	CS	IPV support services (help-seeking group), Com	2,212 m (611 IPV, 1601 Com)	18–59 IPV: (43.89; 9.18), Com: (41.77; 11.35)	CC	PMWI-DI	CTS2	CTS2	PTSD, Depression	PCL-C, CESD
* Ireland et al. (2017)	Australia	CS	Com: LGBTI	287 LGBTI w and m	≥18 (34.8; 11.2)	CC	MMEA (extended 44 items)	MMEA (extended 44 items)	MMEA (extended 44 items)	PTSD, Depression, Anxiety	PCL-C-SF, HAD
* Johnson and Leone (2005)	USA	CS—2nd	Com: NVAWS national survey	4,967 w (81 IT)	18–97 (44.55; 13.89)	CC	NVAWS-CC items		12-items adapted from CTS	PTSD, Depression	IES (adapted), 8-items depression scale (based on SF-36)
Johnson et al. (2014)	USA	CS—2nd	Com: NVAWS national survey	7,782 w 6,908 m	18–97 (46.91; 15.67)	CC	12-items CC-Scale like PMWI		12-items adapted from CTS	Depression	8-items depression scale (based on SF-36)
* Jones (2020)	USA	CS	Prison	337 w	18–65	CC	based on CCMIPV			PTSD	PCL-C
* Jones et al. (2005)	USA	CS	DV services, medical and com	172 w	18–40	CC	PMWI-DI-SF	PMWI-EV-SF, SOSPS		PTSD, Depression, Anxiety	PSDSWB BDI, BAI
* Kapiga et al. (2017)	Tanzania	CS	Com	1,021 w	19–70	CC, EA	WHOMCS-CC, EA items	WHOMCS	WHOMCS	Mental Health	SRQ-20
* Lawrence et al. (2009)	USA	LO	Com: married couples	103 w 103 m	18–55 w: (25; 4.3) m: (26.4; 4.7)	CC	MMEA-RE	MMEA-total		Depression, Anxiety	BDI-II, BAI
* Leone (2011)	USA	CS 2nd	CWHRS abused women subsample	369 w	≥18 (31; 9.62)	CC	PCS			PTSD, Depression, Suicidality	PSS-I, MOS-4 items SI + SA combined 1item
* Levine and Fritz (2016)	Canada	CS	Shelter	51 w	19–58 (37)	CC	CBS-R	CTS2		PTSD, Depression	PCL-C, BDI-II
* Lovestad et al. (2017)	Sweden	CS	Com: population sample	573 w	18–65 (42.7: 13.01)	CC	CBS-isolating control	WHOMCS	WHOMCS	Depression	self-report of 5 DSMIV indicators
* Ludermir et al. (2010)	Brazil	CS	Com: pregnant women from a family health program	1,045 w	18–49	CC	WHOMCS-CC	WHOMCS	WHOMCS	Postnatal Depression	EPDS
* Ludermir et al. (2010)	Brazil	CS	Com: pregnant women from a family health program	1,045 w	18–49	CC	WHOMCS-CC	WHOMCS	WHOMCS	Postnatal Depression	EPDS
Ludermir et al. (2014)	Brazil	CS	Com: pregnant women from a family health program	1,120 w	18–49	CC	WHOMCS-CC	WHOMCS	WHOMCS	Mental Health	SRQ-20
McCauley et al. (2014)	Cote d'Ivoire	CS	Com	953 w	≥18, (37.4)	RC	Based on Miller et al. (2010)			PTSD	HTQ
Mechanic et al. (2000)	USA	CS	DV support services	114 w	19–59 (35; 7.9)	(CC) ST	SBC (PMWI-DI)	PMWI-EV	CTS2	PTSD, Depression	PDS, BDI-II
* Mechanic et al. (2008)	USA	CS	DV support services	413 w	18–62 (34.5; 8.1)	CC, ST	PMWI-DI, SBC	PMWI-EV		PTSD, Depression	PDS, BDI-II
Mutiso et al. (2020)	Kenya	CS	Com: Healthcare services	5,448 w 2,862 m	18–60+	CC	WHOMCS-CC items	WHOMCS	WHOMCS	Mental Health	MINI Plus
Newton (2021)	USA	CS	Com	69 w	≥18 (54.53; 3.19)	CC	PMWI-DI-SF	PMWI-DI-SF	CTS2	PTSD—peritraumatic emotions	CAPS for DSM-IV
* Nielsen et al. (2016)	USA	CS	Com: Coparenting	147 w	20–54 (35.2)	CC	PMWI-DI-SF	PMWI-EV-SF	CTS2	PTSD, Depression	PCL, CESD-SF
* Norwood and Murphy (2012)	USA	CS 2nd	DV support services	216 w	18–70 (34.0, 10.1)	CC	MMEA-RE	MMEA-total	CTS2	PTSD	PCL-C-IPV
* Nnawulezi and Murphy (2019)	USA	CS	DV support services (archival data)	228 w	18–70 (34.23; 10.09)	CC	MMEA-RE	MMEA-total	CTS2	PTSD	PCL-C-IPV
* Ogunbajo et al. (2020)	Nigeria	CS	Com	389 m	≥18	CC	IPV-GBM monitoring controlling	IPV-GBM	IPV-GBM	Depression, Suicidality, Anxiety	CESD SI-1-item, SA-1-item GAD-7
Peltzer et al. (2013)	South Africa	CS	IPV survivors who obtained a protective order	268 w	18–62 (28.8; 8.0)	ST	SVS, 10 items from HARASS	SVAWS	SVAWS	PTSD, Depression Alcohol Use	PCL-C, CESD AUDIT-C
* Petra (2020)	USA	CS	Com	222 w	24–63 (33)	CC	MASIC-CC subscale		WAST	Depression, Anxiety	DASS-21
* Pickover et al. (2017)	USA	CS	IPV survivors, mental health research clinic	284 w	≥18 (37.84; 12.08)	CC	PMWI-DI-SF	PMWI-EV-SF	CTS2	PTSD, Anxiety	LEC, IPV-related DSMIV-TR, ADIS-IV
Postmus et al. (2012)	USA	LO	Com	2,305 w	≥18 (25.8; 6.1)	EA	Any EA in year 1	Any psych. IPV in year 1	Any physic. IPV in year 1	Depression	CIDI-SF
Prospero (2008)	USA	CS	University students	609 (361 w, 248 m)	≥18 (21.44; 4.58)	CC	CBS-R	CTS2	CTS2	Mental Health	SQ
Prospero (2009)	USA	CS	University students	573 (332 w, 241 m)	≥18 (21.4; 4.37)	CC	CBS-R	CTS2	CTS2	Mental Health	SQ
* Prospero and Kim (2009a)	USA	CS	University students	560 (325 w, 235 m)	≥18 (21.4; 4.37)	CC, EA	CBS-R	CTS2	CTS2	Depression, Anxiety	SQ
Prospero and Kim (2009b)	USA	CS	University students	676 (419 w, 257 m)	≥18 (21.6; 4.66)	CC, EA	CBS-R	CTS2	CTS2	Mental Health	SQ
* Reich et al. (2015)	USA	CS	IPV survivors—mixed recruitment	105 w	≥18 (36.94;12.68)	CC	PMWI-DI-SF	PMWI-EV-SF	CTS2	PTSD	CAPS for DSM-IV
* Sackett and Saunders (1999)	USA	CS	DV support services	60 w	≥18 (34.7; 9.1)	CC	PPA-JC	PPA		Depression, Anxiety	BDI 6-item Anxiety Scale
Sauber and O'Brien (2020)	USA	CS	DV support services, community	147 w	≥18 (34.7; 9.1)	EA	SEA-12	ABI	ABI	PTSD, Depression	PCL-C, CESD
Strauss et al. (2019)	USA	CS	University students	357 (290 w, 67 m)	18–25 (18.64; 1.13)	ST	32-item validated ST measure			Alcohol and Drug Use	AUDIT DUDIT
* Street and Arias (2001)	USA	CS	Shelter	63 w	19–64 (32)	CC	PMWI-DI	PMWI-EV	CTS	PTSD, Depression	CMS, SCL-90-R
Stylianou (2018)	USA, Puerto Rico	LO	DV support services	457 w	≥18 (36; 9.15)	EA	SEA-12	ABI-R	ABI-R	Depression	CESD
* Taft et al. (2005)	USA	LO	Partners of IPV perpetrator group program participants	96 w	18–59 (34.0; 8.6)	CC	MMEA-RE	MMEA-total	CTS	PTSD	DIS for DSM-III
* Tiwari et al. (2015)	Hong Kong	CS	DV support services	613 w	≥20	CC	CBS-R (Chinese)	C-AAS		PTSD, Depression	PCL-C (Chinese) BDI-II (Chinese)
Voth Schrag et al. (2019)	USA	CS	University students	435 w	≥18 (27; 9.9)	EA	SEA-12		ABI-R	PTSD, Depression	PCL-5 LEC-5, CESD-SF
* Weaver and Etzel (2003)	USA	CS	DV support services	62 w	≥18 (34; 8.4)	CC	PMWI-DI-SF	PMWI-EV-SF	CTS2	PTSD, Depression *	PDS, BDI-II
Wolf et al. (2018)	Denmark	CS	ST support services, groups	196 w	27–70 (40.82; 6.93)	ST	SBC	PMWI		PTSD	HTQ, TSC-33
* Wolford-Clevenger et al. (2017)	USA	CS	University students	208 (107 w, 101 m)	18–29 (19.61; 11.09)	CC	MMEA-RE	MMEA	CTS2	Depression, Suicidal Ideation	PDSQ
* Wolford-Clevenger and Smith (2017)	USA	CS	Shelter	134 w	19–67 (32.50; 8.21)	CC	CIPRS		CTS2 (modified)	PTSD, Depression, Suicidal Ideation and Attempts	PCL-C, DASS-21 SI: MSSI SA: L-SASI

*Note*. Study type: CS = cross-sectional; LO = longitudinal; 2nd = secondary data analysis; Sample: w = women; m = men; Age: M = mean; SD = standard deviation; IPV = intimate partner violence; DV = domestic violence; PTSD = post-traumatic stress disorder; Type of coercive control: CC = coercive control; EA = economic abuse; RC = reproductive coercion; ST = stalking. Recruitment: Com = community sample (i.e., not specific IPV sample); NVAWS = National Violence Against Women survey (Tjaden & Thoennes, 1999); NISVS = The National Intimate Partner and Sexual Violence Survey (Smith et al., 2017); CWHRS = Chicago Women’s Health Risk Study (Block, 2000); WHES = Women’s Health Effects Study (Ford-Gilboe et al. 2009). IPV Measures: ABI = The Abusive Behavior Inventory (Shepard & Campbell, 1992); ABI-R = Abuse Behavior Inventory–Revised (Postmus et al. 2016b); CAS = Composite Abuse Scale (Hegarty et al.,1999, 2005); CAAS = Chinese Abuse Assessment Screen (Tiwari et al., 2007); CBS = Controlling Behaviors Scale (Graham-Kevan & Archer, 2003a); CBS-R = Controlling Behaviors Scale-Revised (Graham-Kevan & Archer, 2003b); CCMIPV = Coercive Control Measure for IPV (Dutton et al., 2005); CIPRS = Coercion in Intimate Partner Relationships Scale (Dutton et al., 2007); CTS = Conflict Tactics Scale (Straus, 1990); CTS2 = Revised Conflict Tactics Scale (Straus et al., 1996); HARASS = Harassment of battered women (Sheridan, 1998); IPV-GBM = Intimate Partner Violence among Gay and Bisexual Men Scale (Stephenson & Finneran, 2013); MASIC = Mediator’s Assessment of Safety Issues and Concerns (Pokman et al., 2014); MMEA = Multidimensional Measure of Emotional Abuse (Murphy et al., 1999); MMEA-RE restrictive engulfment subscale; PCS = Power and Control Scale (Leone et al., 2007); PMWI, PMWI-SF = Psychological Maltreatment of Women Inventory (Tolman, 1989;^1999^) with PMWI-DI = dominance/isolation subscale, PMWI-EV = emotional/verbal abuse subscale; PPA = Profile of Psychological Abuse (Sacket & Saunders, 1999); SEA-12 = Scale of Economic Abuse-12 (Postmus et al., 2016a); SBC = Stalking Behavior Checklist (Coleman, 1997); SRPS = Sexual Relationship Power Scale (Pulerwitz et al., 2002); SVAWS = Severity of Violence Against Women Scale (Marshall, 1992); SVS = Stalking Victimization Survey (Tjaden & Thoennes, 1999); WAST = Woman Abuse Screening Tool (Brown, 1996); WEB = Women’s Experiences with Battering (Smith et al., 1999); WHOMCS = WHO Multi-country Study on Women's Health and Domestic Violence Against Women (Garcia-Moreno et al., 2005). PTSD: CAPS = Clinician-Administered PTSD Scale (Blake et al., 1990); CMS = The Civilian Mississippi Scale for PTSD (Vreven et al., 1995); DIS = Diagnostic Interview Schedule for DSM-III (Robins et al., 1982); HTQ = Harvard Trauma Questionnaire (Mollica et al., 1992); IES = Impact of Event Scale (Weiss & Marmar, 1997); ITQ = International Trauma Questionnaire (Cloitre et al., 2018); LEC = Life Events Checklist (Gray et al., 2004); LEC-5 = Life Events Checklist for DSM-5 (Weathers et al., 2013a); PCL = PTSD Checklist (Blanchard et al., 1996); PCL-C = PTSD Checklist-Civilian (Weathers et al., 1993); PCL-C-SF = PTSD Checklist-Civilian Short Form (Lang & Stein, 2005); PCL-C-IPV = PTSD Checklist-Civilian Modified to IPV experience (Weathers et al., 1993); PCL-5 = The PTSD Checklist for DSM-5 (Weathers et al., 2013b); PCPTSD = Primary Care Post-Traumatic Stress Disorder Screen (Prins et al., 2003); PDS = The Posttraumatic Diagnostic Scale (Foa et al., 1997); PSDSWB = Posttraumatic Stress Disorder Scale for Battered Women (Saunders, 1994); PSS-I = PTSD Symptom Scale Interview (Foa, et al., 1993a); PSS-SR = PTSD Symptom Scale Self-Report Version (Foa, et al., 1993b); TSC-33 = The Trauma Symptom Checklist (Briere & Runtz, 1989); TSC-26 = The Revised Trauma Symptom Checklist (Krog & Duel, 2003). Depression, Suicidality, Anxiety, Mental Health: ADIS-IV = Anxiety Disorders Interview Schedule-IV (Brown et al., 1994); BAI = Beck Anxiety Inventory (Beck et al., 1988); BDI = Beck Depression Inventory (Beck et al., 1961); BDI-II = Beck Depression Inventory (Beck et al., 1996); BSI = Brief Symptom Inventory (Derogatis, 1993); CESD = Center for Epidemiologic Depression Scale (Radloff, 1977); CESD-SF = CESD Short Form (Andresen et al., 1994); CESD-R = (Eaton et al., 2004); CIDI-SF = International Diagnostic Interview, Short Form (Kessler et al., 1998); DASS-21 = The Depression Anxiety Stress Scale 21 (Lovibond & Lovibond, 1995); EPDS = Edinburgh postnatal depression scale (Cox et al., 1987); GAD-7 = Generalized Anxiety Disorder 7-item (Spitzer et al., 2006); HAD = Hospital Anxiety and Depression Scale (Zigmond & Snaith, 1983); HSCL-25= Hopkins Symptom Checklist (Parloff et al., 1954; Winokur et al., 1984); MINI Plus = International Neuropsychiatric Interview (Sheehan et al., 1997); MOS = Medical Outcome Study (Hays et al., 1995); PDSQ = Psychiatric Diagnostic Screening Questionnaire (Zimmerman & Mattia, 2001); L-SASI = Lifetime-Suicide Attempt Self-Injury Count (Linehan et al., 2006); SA = Suicide Attempt SCL-90-R = Symptom Checklist 90 (Derogatis, 1983); SF-36 = Short-Form Health Survey (Ware & Sherbourne, 1992); SI = Suicidal Ideation; SQ = Symptom Questionnaire (Kellner, 1987); SRQ-20 = Self-reporting Questionnaire (Beusenberg et al.,1994); STAI = State-Trait Anxiety Inventory (Spielberger et al., 1983); Alcohol and Drug Use: AUDIT = Alcohol Use Disorders Identification Test (Saunders et al., 1993); AUDIT-C = Alcohol Consumption Questions (Bush, 1998); CAGE = CAGE questionnaire (Ewing, 1984); DUDIT = Drug Use Disorders Identification test (Stuart et al., 2003); MFDAQ = Monitoring the future drug and alcohol questionnaire (Bachman et al., 2011); NWS = National Women’s Study (Kilpatrick et al., 1997). References for these measures are provided in Supplemental Appendix D.

* Studies included in the meta-analysis.

### Coercive Control Measures

Coercive control was measured with a range of scales and subscales. The domination/isolation subscale of the PMWI-DI (Tolman, 1989) or its short form (PMWI-SF-DI; Tolman, 1999) were most frequently used (*n* = 17). The *Controlling Behaviors Scale-Revised* (Graham-Kevan & Archer, 2003) was the second most frequently used measure (*n* = 7), followed by the *Multidimensional Measure of Emotional Abuse* (MMEA; Murphy et al., 1999) (*n* = 6), particularly the Restrictive Engulfment Subscale (MMEA-RE). Several studies in Non-Western countries used the controlling behaviors questions from the *WHO Multi-country Study on Women’s Health and Domestic Violence Against Women* (Garcia-Moreno et al., 2005)(*n* = 6). Other studies used the power and control questions that were developed for the *National Violence Against Women* survey (Tjaden & Thoennes, 1999)(*n* = 5). Each of the remaining studies used a different scale: *Composite Abuse Scale* (Hegarty et al.,1999, 2005), *Women’s Experiences with Battering* (Smith et al., 1999), *Scale of Power and Control* (Block, 2000), *Power and Control Scale* (Leone et al., 2007), controlling questions from the *Intimate Partner Violence among Gay and Bisexual Men Scale* (IPV-GBM; Stephenson & Finneran, 2013), coercive control subscale of the *Mediator’s Assessment of Safety Issues and Concerns* (Pokman et al., 2014), *Sexual Relationship Power Scale* (Pulerwitz et al., 2002), coercive tactics subscale from the *Coercion in Intimate Partner Relationships Scale* (Dutton et al., 2007), coercive control questions from *The National Intimate Partner and Sexual Violence Survey* (Smith et al., 2017),the Jealous/Control Scale from the *Profile of Psychological Abuse* (Sackett & Saunders, 1999) and a modified version of Dutton et al.’s (2005) *Coercive Control Measure for IPV*. Specific measures of economic abuse, stalking, and reproductive coercion are also identified in Table 1.

### Mental Health Measures

PTSD symptom severity was measured by 31 studies and was most frequently measured with the *PTSD Checklist-Civilian* (Weathers et al., 1993). Depression was measured by 38 studies, the most frequently used measure was the *Center for Epidemiologic Depression Scale* (Radloff, 1977). Notably, only one study (Dokkedahl et al., 2021) measured CPTSD by using the International Trauma Questionnaire (Cloitre et al., 2018). The mental health measures of all studies included in the qualitative synthesis are summarized in Table 1.

### Quality Assessment

The reviewers initially obtained a 90% agreement and any conflicts in the quality assessment that remained were discussed and resolved by consensus. The most common risk of bias was that studies did not clearly report or address potential confounds. A summary table of the quality assessment for all studies included in the qualitative synthesis is included in Supplemental Appendix C. The appraisal tool does not offer guidance about cut-off scores to assess the overall level of risk of bias for each study and we could therefore not establish the overall risk of bias for each study (Munn et al., 2020).

## Meta-Analyses

A total of 45 studies with 107 effect sizes addressed associations involving coercive control with PTSD and depression and were included across a series of random effects meta-analyses. The numbers of studies, effect size estimates (weighted mean correlations), 95% CIs, and heterogeneity statistic (*I*^2^) for these meta-analyses are summarized in Table 2. Forest plots for each meta-analysis are included in Supplemental Appendix E.

**Table 2.** Results of Random-Effects Meta-Analyses.

Association	Studies *k*	Effect Sizes *k*				*r*		95% CI	*I*^2^ (%)
		Total	Women	Men	Both	Range	Mean		
PTSD									
Coercive control	30	31	28	2	1	−.08 to .56	.32*	[.28, .37]	71.77*
Psychological IPV	19	19	17	1	1	−.15 to .64	.34*	[.25, .42]	88.48*
Depression									
Coercive control	35	38	31	3	4	−.09 to .59	.27*	[.22, .31]	87.20*
Psychological IPV	18	19	15	2	2	−.08 to .60	.33*	[.26, .40]	85.45*

*Note*. Coercive control includes economic abuse, stalking, reproductive coercion. IPV = intimate partner violence; PTSD = post-traumatic stress disorder.

* *p* ≤ .001

### Coercive Control, PTSD, and Depression

The meta-analyses involving coercive control and PTSD identified a significant moderate positive association (*r* = .32; 95% CI [.28, .37]) when pooled across studies, with high heterogeneity, *Q*(20) = 97.62, *I*^2^ = 79.51%, *p* < .001. The meta-analyses involving coercive control and depression showed a significant moderate positive correlation (*r* = .27; [.22, .31]) when pooled across studies, with high heterogeneity, *Q*(37) = 289.02, *I*^2^ = 87.20%, *p* < .001).

#### Subgroup analyses

Subgroup analyses for studies addressing associations involving coercive control with PTSD and depression were performed to examine sources of heterogeneity. These analyses included comparisons according to (a) types of coercive control measure (general coercive control measures vs. specific economic abuse, stalking and reproductive coercion measures) and (b) study settings (domestic violence support services/shelters vs. community). The inspection of the 95% CIs showed a statistically significant difference in the strength of mean correlations between coercive control and PTSD according to study settings, with a stronger pooled association observed in studies of domestic violence support services/shelters settings (*r* = .40; 95% CI [.35, .45]), when compared to community settings (*r* = .26; [.16, .35]). There were no other significant effects. Findings for all performed subgroup analyses including pooled correlations, 95% CIs and heterogeneity (*I*^2^) of studies are summarized in Supplemental Appendix F.

### Psychological IPV, PTSD, and Depression

The random-effects meta-analysis showed a significant moderate positive association between psychological IPV and PTSD (*r* = .34; 95% CI [.25, .42]) with high heterogeneity between studies, *Q*(18) = 156.23, *I*^2^ = 88.48%, *p* < .001. The random-effects meta-analysis between psychological IPV and depression showed a significant moderate positive association between psychological IPV and depression (*r* = .33; 95% CI [.26, .40]) with high heterogeneity between studies, *Q*(18) = 124.36, *I*^2^ = 85.45%, *p* < .001. Inspection of the 95% CIs suggests that there are no statistically significant differences in the associations between coercive control and psychological IPV in relation to PTSD and depression (see Table 2).

### Publication Bias

All analyses were found to be robust against the risk of publication bias. The results of the Duval and Tweedie’s (2000) trim and fill test, the classic fail-safe *N* test (Rosenthal, 1979), and Orwin’s (1983) fail-safe *N* test for each meta-analysis are summarized in Supplemental Appendix G.

## Discussion

This review examined the mental health implications of coercive control and identified moderate associations with measures of PTSD and depression symptom severity, when considered across all available studies. The overall strength of these associations were comparable to those involving broader measures of psychological IPV with both PTSD and depression. Furthermore, the strength of the associations were comparable to those for physical IPV and combined IPV found in previous meta-analyses. For instance, Spencer et al.’s (2019) large meta-analysis found small to moderate correlations between physical IPV and PTSD (*r* = .34), as well as depression (*r* = .25).

It was unexpected that associations of coercive control with PTSD and depression would not be clearly stronger than associations involving other types of IPV (including broader measures of psychological IPV), and there are several possible explanations for this. First, considering the difficulties of distinguishing coercive control from broader dimensions of psychological IPV in psychometric measures, construct overlap remains likely and could explain similar effects (Dutton & Goodman, 2005). Relatedly, the psychometric measures and subscales that were used to measure coercive control in this review may not have fully captured whether a behavior occurred in the context of coercive control. For instance, many measures may not fully capture whether respondents experience a threat that is embedded in a chronic pattern of power and control (Johnson, 2008). Second, the chronic pattern of terror and the effects of entrapment that characterize coercive control may be difficult to quantify, and they may not be as clearly measured in psychometric instruments compared to the occurrence of specific behaviors (Dokkedahl et al., 2019). Nevertheless, the similar strength of links involving coercive control and broader dimensions of psychological IPV and physical IPV with mental health symptoms reported in previous meta-analyses, suggests that these dimensions of coercive control are just as important and detrimental as physical IPV.

It was not possible to investigate whether coercive control was associated with CPTSD symptom severity, as only one eligible study measured CPTSD (Dokkedahl et al., 2021). This study reported a small positive correlation (*r* = .23) between coercive control and CPTSD, with stronger links also observed between broader psychological IPV and CPTSD when compared to physical IPV in a shelter sample of 147 women. This dearth of empirical studies, along with Dokkedahl et al.’s initial findings and emerging evidence from qualitative studies (Baird et al., 2019; Salter et al., 2020), as well as strong conceptual reasons for expecting CPTSD symptoms to develop in response to coercive control (Cloitre, 2021; Herman, 1992; WHO, 2019), suggests an urgent need for more research into the relationship between coercive control and CPTSD.

Finally, combined subgroup analyses of economic abuse, reproductive coercion, and stalking did not indicate any meaningful differences compared to general coercive control, but these types of coercive control were not reviewed separately and may have unique impacts that could not be investigated in this review. Notably, subgroup analyses indicated that the associations between coercive control and PTSD were stronger in domestic violence support service/shelter settings compared to community settings, suggesting that the incidence and/or impact of coercive control may be greater in domestic violence crisis response settings.

### Limitations

The present study had several limitations, and the findings have to be interpreted accordingly. First, the findings included in the meta-analyses were cross-sectional and a direct causal link between coercive control and mental health could not be established. Second, the overall level of quality in the body of evidence could not be assessed with certainty and clear conclusions about the quality of the evidence could not be drawn. Third, most of the data in the included studies were derived from self-report measures and may be subject to under or overreporting. We also limited our search to English language reports, which has limited the access to evidence from non-English speaking countries and cultures. Moreover, the majority of studies used symptom severity measures. Only 7.35% of the studies included in the qualitative synthesis, and only 4.44% of the studies included in the meta-analyses used diagnostic instruments (Beck et al., 2011; Mutiso et al., 2020; Newton, 2021; Pickover et al., 2017; Reich et al. 2015). Thus, there was less clear evidence for a direct link between coercive control and mental health diagnoses. Finally, high heterogeneity suggests that other study features that could not be examined in this review may account for this variability. For instance, 76% of the studies focused solely on women, and only three studies focused on men and two on gender diverse populations and subgroup analyses could not be performed. Differences in female, male, and gender diverse populations may help to explain some of the heterogeneity.

## Conclusion

Despite these limitations, the findings of this review provide important evidence for the mental health implications of coercive control exposure. This was the first meta-analysis that examined the associations involving coercive control and mental health. Results indicate that coercive control exposure is moderately associated with PTSD and depression symptom severity. The strength of these associations were comparable to those involving measures of broader psychological IPV in the present meta-analyses, and to those for physical IPV found in previous meta-analyses. Key findings are summarized in Table 3.

**Table 3.** Summary of Critical Findings.

• Coercive control exposure was moderately associated with PTSD and depression symptom severity • The strength of these associations was comparable to those involving measures of broader psychological IPV The strength of these associations was comparable to those for physical IPV found in previous meta-analyses

*Note*. IPV = intimate partner violence; PTSD = post-traumatic stress disorder.

## Implications

These findings have important implications for clinical practice research, policy, and legislation.

### Implications for Clinical Practice

This meta-analysis provided evidence that coercive control exposure is linked to PTSD and depression, suggesting that coercive control exposure can have long-term mental health implications and that individuals who have been exposed to coercive control would likely benefit from psychological support. However, presently most IPV interventions focus on safety and crisis management (Neave et al., 2016). Subgroup analyses indicated a stronger link between coercive control and PTSD in domestic violence service/shelter settings suggesting that there is a need to include short-term mental health support in crisis response services. Moreover, evidence-based interventions are urgently needed to support long-term recovery, and clinicians need to be trained and supported so that they can provide effective short- and long-term care.

### Implications for Research

First, the complexity of the coercive control construct and the difficulty to fully and distinctly capture it in most commonly used psychometric measures suggests the need to use more comprehensive measures of coercive control in primary studies. Equally, qualitative research approaches may be well suited to address the nuances in behaviors, such as verbal threats, to determine if they occur within the context of situational couple violence or coercive control. Third, this review identified a lack of empirical studies that have investigated the relationship between coercive control and CPTSD, and more research is needed. Finally, most of the studies were conducted in developed countries and predominantly focused on women in heterosexual relationships. More primary studies in developing countries, and studies with gender diverse samples are needed.

### Implications for Policy and Legislation

The findings highlight that policy makers and legislators need to consider the mental health impacts of coercive control when implementing policies and legislations surrounding the criminalization of coercive control, and to provide funding for trauma-informed mental health services that support the long-term recovery of those who have been exposed to coercive control. The implications for clinical practice, research, and policy and legislation are summarized in Table 4.

**Table 4.** Implications for Clinical Practice, Research, Policy, and Legislation.

Implications for Clinical Practice • Coercive control exposure is linked to PTSD and depression, suggesting long-term mental health implications that require mental health support • Trauma-informed interventions are needed to support long-term recovery • A stronger link between coercive control and PTSD in domestic violence service/shelter settings suggests a need to include short-term trauma-informed mental health care in crisis response services • Clinicians need to be trained and supported so that they can provide effective short- and long-term care. Implications for Research • Coercive control is difficult to capture in most commonly used psychometric measures and more comprehensive measures of coercive control need to be used in primary studies • Research into the development of more specific coercive control measures is needed • Qualitative research approaches may be well suited to address the nuances in behaviors, such as verbal threats, to determine if they occur within the context of situational couple violence or coercive control. • There is lack of empirical studies that have investigated the relationship between coercive control and CPTSD, and more research is needed • More research in developing countries is needed • More research with gender diverse samples is needed Implications for Policy and Legislation • The mental health impacts of coercive control need to be considered in policies and legislations surrounding the criminalization of coercive control Funding for trauma-informed mental health care that supports the long-term recovery of those who have been exposed to coercive control is needed

*Note*. IPV = intimate partner violence; PTSD = post-traumatic stress disorder; CPTSD = complex post-traumatic stress disorder.

## Supplemental Material

sj-docx-1-tva-10.1177_15248380231162972 – Supplemental material for The Trauma and Mental Health Impacts of Coercive Control: A Systematic Review and Meta-Analysis Click here for additional data file. Supplemental material, sj-docx-1-tva-10.1177_15248380231162972 for The Trauma and Mental Health Impacts of Coercive Control: A Systematic Review and Meta-Analysis by Susanne Lohmann, Sean Cowlishaw, Luke Ney, Meaghan O’Donnell and Kim Felmingham in Trauma, Violence, & Abuse
