# Supplementary material for: The Trauma and Mental Health Impacts of Coercive Control: A Systematic Review and Meta-Analysis
Source: Trauma Violence Abuse. 2023 Apr 13;25(1):630–47. doi: 10.1177/15248380231162972 (PMC10666508; doi:10.1177/15248380231162972)
Supplement: sj-docx-1-tva-10.1177_15248380231162972 – Supplemental material for The Trauma and Mental Health Impacts of Coercive Control: A Systematic Review and Meta-Analysis [file sj-docx-1-tva-10.1177_15248380231162972.docx]

The Trauma and Mental Health Impacts of Coercive Control: A Systematic Review and Meta-Analysis

Supplementary Material

Appendix A: Search syntax PsycINFO, Medline, CINAHL and Scopus

Appendix B: List of Excluded Reports and Reasons for Exclusion

Appendix C: Quality Assessment for all 68 Reports Included in the Qualitative Synthesis Including 45 Included in the Meta-analyses

Appendix D: Supplementary Reference List of Measures

Appendix E: Forest Plots for Meta-analyses involving coercive control and PTSD, and coercive control and depression

Appendix F: Subgroup Analyses for Coercive Control, PTSD and Depression

Appendix G: Risk of Publication Bias

# Appendix A

Search Syntax for each Database Search

*PsycINFO Search Syntax*

|  | Search Algorithm |
| --- | --- |
| 1. Exposure 1 | (coerc* ADJ6 control*) or coercion or coercive or "intimate terror*" or threat or humiliation or intimidation or "psychological abuse" or "psychological violence" or "psychological aggression" or "emotional abuse" or "monitoring" or "entrapment" |
| 2. Exposure 2 | "intimate partner violence" or IPV or IPA or "partner abuse" or "spouse abuse" or "domestic violence" or "domestic abuse" or "battered" |
| 3. Outcome | "mental health" or "mental disorder*" or "mental* ill*" or psychopathology or trauma* or posttraumatic or PTSD or CPTSD or depress* or anxi* or panic or phobia or psychosis or "psychotic" or schizophrenia or "substance abuse" or "substance use" or "alcohol abuse" or "alcohol use" or alcoholism or "drug abuse" or "drug use" or suicid* or "self harm" or shame or "emotional regulation" or "emotional dysregulation" or "affect regulation" or "affect dysregulation" |
| 4. | 1 and 2 and 3 |

Search completed om 11/5/21: 1192 records retrieved

*Medline (Ovid) Search Syntax*

|  | Search Algorithm |
| --- | --- |
| 1. Exposure 1 | (coerc* ADJ6 control*) or coercion or coercive or "intimate terror*" or threat or humiliation or intimidation or "psychological abuse" or "psychological violence" or "psychological aggression" or "emotional abuse" or "monitoring" or "entrapment" |
| 2. Exposure 2 | "intimate partner violence" or IPV or IPA or "partner abuse" or "spouse abuse" or "domestic violence" or "domestic abuse" or "battered" |
| 3. Outcome | "mental health" or "mental disorder*" or "mental* ill*" or psychopathology or trauma* or posttraumatic or PTSD or CPTSD or depress* or anxi* or panic or phobia or psychosis or "psychotic" or schizophrenia or "substance abuse" or "substance use" or "alcohol abuse" or "alcohol use" or alcoholism or "drug abuse" or "drug use" or suicid* or "self harm" or shame or "emotional regulation" or "emotional dysregulation" or "affect regulation" or "affect dysregulation" |
| 4. | 1 and 2 and 3 |
| Result | Limited to English language (cannot limit for peer-reviewed in Medline) |

Search completed om 11/5/21: 1082 records retrieved

*CINAHL* *Search Syntax*

|  | Search Algorithm |
| --- | --- |
| 1. Exposure 1 | (coerc* N6 control*) OR coercion or coercive OR "intimate terror*" OR threat OR humiliation OR intimidation OR "psychological abuse" OR "psychological violence" OR "psychological aggression" OR "emotional abuse" OR "monitoring" OR "entrapment" |
| 2. Exposure 2 | "intimate partner violence" OR IPV OR IPA OR "partner abuse" OR "spouse abuse" OR "domestic violence" OR "domestic abuse" OR "battered" |
| 3. Outcome | "mental health" OR "mental disorder*" OR "mental* ill*" OR psychopathology OR trauma* OR posttraumatic OR PTSD OR CPTSD OR depress* OR anxi* OR panic OR phobia OR psychosis OR "psychotic" OR schizophrenia OR "substance abuse" OR "substance use" OR "alcohol abuse" OR "alcohol use" OR alcoholism OR "drug abuse" OR "drug use" or suicid* or "self harm" or shame or "emotional regulation" or "emotional dysregulation" OR "affect regulation" OR "affect dysregulation" |
| 4. | 1 and 2 and 3 |
| Result | Limited to English language, peer-reviewed journals |

Search completed om 11/5/21: 758 records retrieved

*Scopus* *Search Syntax*

|  | Search Algorithm |
| --- | --- |
| 1. Exposure 1 | (coerc* W/6 control*) OR coercion or coercive OR "intimate terror*" OR threat OR humiliation OR intimidation OR "psychological abuse" OR "psychological violence" OR "psychological aggression" OR "emotional abuse" OR "monitoring" OR "entrapment" |
| 2. Exposure 2 | "intimate partner violence" OR IPV OR IPA OR "partner abuse" OR "spouse abuse" OR "domestic violence" OR "domestic abuse" OR "battered" |
| 3. Outcome | "mental health" OR "mental disorder*" OR "mental* ill*" OR psychopathology OR trauma* OR posttraumatic OR PTSD OR CPTSD OR depress* OR anxi* OR panic OR phobia OR psychosis OR "psychotic" OR schizophrenia OR "substance abuse" OR "substance use" OR "alcohol abuse" OR "alcohol use" OR alcoholism OR "drug abuse" OR "drug use" or suicid* or "self harm" or shame or "emotional regulation" or "emotional dysregulation" OR "affect regulation" OR "affect dysregulation" |
| 4. | 1 and 2 and 3 |
| Result | Limited to English language |

Search completed om 11/5/21: 2016 records retrieved, after limiting to English language and excluding books and book chapters 1900 results were retrieved

# Appendix B

Reports that did not Meet the Inclusion Criteria and Reasons for Exclusion

*Table B1: Excluded Reports*

| **Study** | **Title** | **Journal** | **Volume** | **Issue** | **Pages** | **DOI** | **Exclusion Reason** |
| --- | --- | --- | --- | --- | --- | --- | --- |
| Abass 2018 | Association between domestic violence and depression among women attending primary health care center in Al-hilla city | Indian Journal of Public Health Research and Development | 9 | 12 | 971-975 | 10.5958/0976-5506.2018.01975.7 | Exclusion reason:Wrong study design |
| Abbaszadeh 2011 | Violence during pregnancy and postpartum depression | Pakistan Journal of Medical Sciences | 27 | 1 | 177-181 |  | Exclusion reason: No coercive control measure |
| Abbott 1995 | Domestic violence against women. Incidence and prevalence in an emergency department population | JAMA | 273 | 22 | 1763-7 |  | Exclusion reason: No coercive control measure |
| Adebowale 2020 | The association between intimate partner violence, psychiatric morbidity amongst pregnant women and partner alcohol use in southern Nigeria | African Journal of Primary Health Care & Family Medicine | 12 | 1 | e1-e7 | <https://dx.doi.org/10.4102/phcfm.v12i1.2226> | Exclusion reason: Wrong age group; |
| Agardh 2012 | The invisible suffering: Sexual coercion, interpersonal violence, and mental health-A cross-sectional study among university students in South-Western Uganda | PLoS ONE Vol 7(12), 2012, ArtID e51424 | 7 | 12 |  | <http://dx.doi.org/10.1371/journal.pone.0051424> | Exclusion reason: No coercive control measure |
| Ahmadabadi 2019 | Intimate partner violence in emerging adulthood and subsequent substance use disorders: Findings from a longitudinal study | Addiction | 114 | 7 | 1264-1273 | <http://dx.doi.org/10.1111/add.14592> | Exclusion reason: Does not report any form of coercive control separately from psychological IPV |
| Ahmadabadi 2020 | Intimate partner violence and subsequent depression and anxiety disorders | Social Psychiatry & Psychiatric Epidemiology | 55 | 5 | 611-620 | <https://dx.doi.org/10.1007/s00127-019-01828-1> | Exclusion reason: Does not report any form of coercive control separately from psychological IPV |
| Ajdukovic 2009 | Family violence and health among elderly in croatia | Journal of Aggression, Maltreatment and Trauma | 18 | 3 | 261-279 | 10.1080/10926770902835873 | Exclusion reason: No differentiation between types of abuse |
| Al-Modallal 2012 | Psychological partner violence and women's vulnerability to depression, stress, and anxiety | International Journal of Mental Health Nursing | 21 | 6 | 560-566 | <http://dx.doi.org/10.1111/j.1447-0349.2012.00826.x> | Exclusion reason: Wrong age group |
| Albright 2019 | Intimate partner violence among postsecondary students with military experience | Traumatology | 25 | 1 | 58-65 | <http://dx.doi.org/10.1037/trm0000172> | Exclusion reason: No coercive control measure |
| Alexander 2016 | Reproductive coercion, sexual risk behaviours and mental health symptoms among young low-income behaviourally bisexual women: Implications for nursing practice | Journal of Clinical Nursing | 25 | 23-24 | 3533-3544 | <http://dx.doi.org/10.1111/jocn.13238> | Exclusion reason: No meaningful outcome measures |
| Ali 1999 | Emotional abuse as a precipitating factor for depression in women | Journal of Emotional Abuse | 1 | 4 | 1-13 | 10.1300/J135v01n04_01 | Exclusion reason: Violence not (clearly) IPV |
| Ali 2013 | Intimate partner violence and mental health effects: A population-based study among married women in Karachi, Pakistan | International Journal of Behavioral Medicine | 20 | 1 | 131-139 | <http://dx.doi.org/10.1007/s12529-011-9201-6> | Exclusion reason: No coercive control measure |
| Belay 2019 | Intimate partner violence and maternal depression during pregnancy: A community-based cross-sectional study in Ethiopia | PLoS ONE Vol 14(7), 2019, ArtID e0220003 | 14 | 7 |  | <http://dx.doi.org/10.1371/journal.pone.0220003> | Exclusion reason: Wrong age group |
| Bernstein 2016 | Intimate partner violence experienced by HIV-infected pregnant women in South Africa: A cross-sectional study | BMJ Open | 6 | 8 |  | 10.1136/bmjopen-2016-011999 | Exclusion reason: No meaningful outcome measures |
| Beydoun 2010 | Intimate partner violence as a risk factor for postpartum depression among Canadian women in the Maternity Experience Survey | Annals of Epidemiology | 20 | 8 | 575-583 | 10.1016/j.annepidem.2010.05.011 | Exclusion reason: Wrong age group |
| Blabey 2009 | Experience of a controlling or threatening partner among mothers with persistent symptoms of depression | American Journal of Obstetrics and Gynecology | 201 | 2 | 173.e1-173.e9 | 10.1016/j.ajog.2009.04.025 | Exclusion reason: No coercive control measure |
| Blasco-Ros 2010 | Recovery from depressive symptoms, state anxiety and post-traumatic stress disorder in women exposed to physical and psychological, but not to psychological intimate partner violence alone: A longitudinal study | BMC Psychiatry Vol 10 2010, ArtID 98 | 10 |  |  | <http://dx.doi.org/10.1186/1471-244X-10-98> | Exclusion reason: No coercive control measure; |
| Bonomi 2009 | Intimate partner violence in Latina and non-Latina women | American Journal of Preventive Medicine | 36 | 1 | 43-48 | <http://dx.doi.org/10.1016/j.amepre.2008.09.027> | Exclusion reason: No coercive control measure |
| Bonomi 2009 | Medical and psychosocial diagnoses in women with a history of intimate partner violence | Archives of Internal Medicine | 169 | 18 | 1692-7 | <https://dx.doi.org/10.1001/archinternmed.2009.292> | Exclusion reason: No coercive control measure |
| Brewer 2018 | Intimate partner violence, health, sexuality, and academic performance among a national sample of undergraduates | Journal of American College Health | 66 | 7 | 683-692 | <http://dx.doi.org/10.1080/07448481.2018.1454929> | Exclusion reason: No coercive control measure |
| Bulut 2017 | The relationship between postpartum depression and intimate partner violence | Journal of Clinical and Analytical Medicine | 8 | 2 | 168-171 | 10.4328/JCAM.4801 | Exclusion reason: No coercive control measure |
| Calvete 2007 | Cognitive and coping mechanisms in the interplay between intimate partner violence and depression | Anxiety, Stress & Coping: An International Journal | 20 | 4 | 369-382 | <http://dx.doi.org/10.1080/10615800701628850> | Exclusion reason: Duplicate publication |
| Calvete 2008 | Coping as a mediator and moderator between intimate partner violence and symptoms of anxiety and depression | Violence Against Women | 14 | 8 | 886-904 | <http://dx.doi.org/10.1177/1077801208320907> | Exclusion reason: Does not report any form of coercive control separately from psychological IPV |
| Calvete 2007 | Intimate partner violence and depressive symptoms in women: Cognitive schemas as moderators and mediators | Behaviour Research and Therapy | 45 | 4 | 791-804 | <http://dx.doi.org/10.1016/j.brat.2006.07.006> | Exclusion reason: No meaningful outcome measures |
| Campbell 1997 | Mental and physical health effects of intimate partner violence on women and children | Psychiatric Clinics of North America | 20 | 2 | 353-374 | <http://dx.doi.org/10.1016/S0193-953X%2805%2970317-8> | Exclusion reason: Wrong study design   ; Susanne Lohmann (2021-05-28 19:46:31)(Screen): voted yes because it also addresses mental health consequences for women not only children; |
| Carey 2019 | Forms of Intimate Partner Rape Experienced by Latinas With and Without Posttraumatic Stress Disorder | Partner Abuse | 10 | 1 | 59-76 | 10.1891/1946-6560.10.1.59 | Exclusion reason: No coercive control measure |
| Carlson 2003 | Childhood and adult abuse among women in primary health care: Effects on mental health | Journal of Interpersonal Violence | 18 | 8 | 924-941 | <http://dx.doi.org/10.1177/0886260503253882> | Exclusion reason: No coercive control measure |
| Cascardi 1995 | Characteristics of women physically abused by their spouses and who seek treatment regarding marital conflict | Journal of Consulting and Clinical Psychology | 63 | 4 | 616-623 | <http://dx.doi.org/10.1037/0022-006X.63.4.616> | Exclusion reason: No differentiation between types of abuse |
| Cations 2021 | Impact of Historical Intimate Partner Violence on Wellbeing and Risk for Elder Abuse in Older Women | American Journal of Geriatric Psychiatry |  |  |  | 10.1016/j.jagp.2020.12.026 | Exclusion reason: No differentiation between types of abuse |
| Cavanaugh 2011 | Prevalence and correlates of suicidal behavior among adult female victims of intimate partner violence | Suicide & Life-Threatening Behavior | 41 | 4 | 372-383 | 10.1111/j.1943-278X.2011.00035.x | Exclusion reason: No coercive control measure |
| Certain 2008 | Domestic abuse during the previous year in a sample of postpartum women | Journal of Obstetric, Gynecologic, & Neonatal Nursing: Clinical Scholarship for the Care of Women, Childbearing Families, & Newborns | 37 | 1 | 35-41 | <http://dx.doi.org/10.1111/j.1552-6909.2007.00200.x> | Exclusion reason: No differentiation between types of abuse |
| Chan 2008 | Prevalence of dating partner violence and suicidal ideation among male and female university students worldwide | Journal of Midwifery & Women's Health | 53 | 6 | 529-537 | <http://dx.doi.org/10.1016/j.jmwh.2008.04.016> | Exclusion reason: No coercive control measure |
| Chan 2011 | Female victimization and intimate partner violence after the May 12, 2008, Sichuan earthquake | Violence and Victims | 26 | 3 | 364-376 | 10.1891/0886-6708.26.3.364 | Exclusion reason: No coercive control measure |
| Chandra 2009 | Women reporting intimate partner violence in India: Associations with PTSD and depressive symptoms | Archives of Women's Mental Health | 12 | 4 | 203-209 | <http://dx.doi.org/10.1007/s00737-009-0065-6> | Exclusion reason: No coercive control measure; |
| Choi 2019 | Intimate Partner Violence Victimization, Social Support, and Resilience: Effects on the Anxiety Levels of Young Mothers | Journal of Interpersonal Violence |  |  | 8.86261E+14 | <https://dx.doi.org/10.1177/0886260519888532> | Exclusion reason: Wrong age group |
| Christopher 2012 | College women's experiences of intimate partner violence: Exploring mental health issues | NASPA Journal About Women in Higher Education | 5 | 2 | 166-183 | 10.1515/njawhe-2012-1116 | Exclusion reason: No coercive control measure; |
| Coker 2002 | Social support protects against the negative effects of partner violence on mental health | Journal of Women's Health & Gender-Based Medicine | 11 | 5 | 465-476 | <http://dx.doi.org/10.1089/15246090260137644> | Exclusion reason: Does not report any form of coercive control separately from psychological IPV |
| Comecanha 2017 | Clinically speaking, psychological abuse matters | Comprehensive Psychiatry | 73 |  | 120-126 | <http://dx.doi.org/10.1016/j.comppsych.2016.11.015> | Exclusion reason: Does not report any form of coercive control separately from psychological IPV |
| Cook 2006 | Beyond Frequency and Severity: Development and Validation of the Brief Coercion and Conflict Scales | Violence Against Women | 12 | 11 | 1050-1072 | <http://dx.doi.org/10.1177/1077801206293333> | Exclusion reason: No coercive control measure |
| Costa 2015 | Intimate partner violence and health-related quality of life in European men and women: Findings from the DOVE study | Quality of Life Research: An International Journal of Quality of Life Aspects of Treatment, Care & Rehabilitation | 24 | 2 | 463-471 | <http://dx.doi.org/10.1007/s11136-014-0766-9> | Exclusion reason: No coercive control measure |
| Alsaker 2008 | Health-related quality of life among abused women one year after leaving a violent partner | Social Indicators Research | 86 | 3 | 497-509 | <http://dx.doi.org/10.1007/s11205-007-9182-7> | Exclusion reason: No differentiation between types of abuse |
| Alsaker 2006 | Low health-related quality of life among abused women | Quality of Life Research: An International Journal of Quality of Life Aspects of Treatment, Care & Rehabilitation | 15 | 6 | 959-965 | <http://dx.doi.org/10.1007/s11136-006-0046-4> | Exclusion reason: No coercive control measure |
| Alvarez-delArco 2013 | Violence in adulthood and mental health: Gender and immigrant status | Journal of Interpersonal Violence | 28 | 11 | 2203-2222 | <http://dx.doi.org/10.1177/0886260512475310> | Exclusion reason: No coercive control measure |
| Amini 2019 | Mental health and social function among women subjected to intimate partner violence: A cross-sectional study | Acta Medica Iranica | 57 | 9 | 544-548 | 10.18502/acta.v57i9.2638 | Exclusion reason: No coercive control measure |
| Anderson 2017 | Sexual minority status and interpersonal victimization in college men | Psychology of Sexual Orientation and Gender Diversity | 4 | 1 | 130-136 | <http://dx.doi.org/10.1037/sgd0000204> | Exclusion reason: No meaningful outcome measures |
| Ansara 2011 | Psychosocial consequences of intimate partner violence for women and men in Canada | Journal of Interpersonal Violence | 26 | 8 | 1628-1645 | 10.1177/0886260510370600 | Exclusion reason: No coercive control measure |
| Anuk 2018 | The association of experience of violence and somatization, depression, and alexithymia: A sample of women with medically unexplained symptoms in Turkey | Archives of Women's Mental Health | 21 | 1 | 93-103 | <http://dx.doi.org/10.1007/s00737-017-0762-5> | Exclusion reason: Violence not (clearly) IPV |
| Arias 1999 | Psychological abuse: Implications for adjustment and commitment to leave violent partners | Violence and Victims | 14 | 1 | 55-67 | <http://dx.doi.org/10.1891/0886-6708.14.1.55> | Exclusion reason: Does not report any form of coercive control separately from psychological IPV; |
| Avant 2011 | Psychological abuse and posttraumatic stress symptoms in college students | Journal of Interpersonal Violence | 26 | 15 | 3080-3097 | <http://dx.doi.org/10.1177/0886260510390954> | Exclusion reason: No coercive control measure; |
| Aye 2020 | Domestic violence victimisation and its association with mental distress: A cross-sectional study of the Yangon Region, Myanmar | BMJ Open | 10 | 9 |  | 10.1136/bmjopen-2020-037936 | Exclusion reason: No differentiation between types of abuse; |
| Babcock 2008 | Intimate partner abuse and PTSD symptomatology: Examining mediators and moderators of the abuse-trauma link | Journal of Family Psychology | 22 | 6 | 809-818 | <http://dx.doi.org/10.1037/a0013808> | Exclusion reason: Does not report any form of coercive control separately from psychological IPV; |
| Babcock 2013 | Factors contributing to ongoing intimate partner abuse: Childhood betrayal trauma and dependence on one's perpetrator | Journal of Interpersonal Violence | 28 | 7 | 1385-1402 | <http://dx.doi.org/10.1177/0886260512468248> | Exclusion reason: No coercive control measure |
| Baldry 2003 | "Stick and stones hurt my bones but his glance and words hurt more": The impact of physiological abuse and physical violence by current and former partners on battered women in Italy | The International Journal of Forensic Mental Health | 2 | 1 | 47-57 | <http://dx.doi.org/10.1080/14999013.2003.10471178> | Exclusion reason: Does not report any form of coercive control separately from psychological IPV; |
| Bandara 2020 | Domestic violence and self-poisoning in Sri Lanka | Psychological Medicine |  |  | 1-9 | <https://dx.doi.org/10.1017/S0033291720002986> | Exclusion reason: Violence not (clearly) IPV |
| Banyard 2011 | The impact of interpersonal violence in adulthood on women's job satisfaction and productivity: The mediating roles of mental and physical health | Psychology of Violence | 1 | 1 | 16-28 | <http://dx.doi.org/10.1037/a0021691> | Exclusion reason: No coercive control measure |
| BarcelonadeMendoza 2018 | Experiences of intimate partner and neighborhood violence and their association with mental health in pregnant women | Journal of Interpersonal Violence | 33 | 6 | 938-959 | <http://dx.doi.org/10.1177/0886260515613346> | Exclusion reason: No coercive control measure |
| Barros-Gomes 2019 | The role of depression in the relationship between psychological and physical intimate partner violence | Journal of Interpersonal Violence | 34 | 18 | 3936-3960 | <http://dx.doi.org/10.1177/0886260516673628> | Exclusion reason: No coercive control measure |
| Bartlett 2018 | Intimate partner violence and disordered eating among male and female veterans | Psychiatry Research | 260 |  | 98-104 | <http://dx.doi.org/10.1016/j.psychres.2017.11.056> | Exclusion reason: No coercive control measure |
| Bauer 2000 | Prevalence and determinants of intimate partner abuse among public hospital primary care patients | Journal of General Internal Medicine | 15 | 11 | 811-7 |  | Exclusion reason: No coercive control measure |
| Beeble 2009 | Main, mediating, and moderating effects of social support on the well-being of survivors of intimate partner violence across 2 years | Journal of Consulting and Clinical Psychology | 77 | 4 | 718-729 | <http://dx.doi.org/10.1037/a0016140> | Exclusion reason: No meaningful outcome measures |
| Beeble 2011 | The impact of neighborhood factors on the well-being of survivors of intimate partner violence over time | American Journal of Community Psychology | 47 | 3-4 | 287-306 | <http://dx.doi.org/10.1007/s10464-010-9398-6> | Exclusion reason: Does not report any form of coercive control separately from psychological IPV |
| Cowden 2019 | Forgiveness moderates relations between psychological abuse and indicators of psychological distress among women in romantic relationships | South African Journal of Science | 115 | 11-12 |  | 10.17159/sajs.2019/6353 | Exclusion reason: Does not report any form of coercive control separately from psychological IPV |
| Craner 2020 | Partner Abuse Among Treatment-Seeking Individuals with Chronic Pain: Prevalence, Characteristics, and Association with Pain-Related Outcomes | Pain Medicine | 21 | 11 | 2789-2798 | <https://dx.doi.org/10.1093/pm/pnaa126> | Exclusion reason: No differentiation between types of abuse |
| Craparo 2014 | Intimate partner violence: Relationships between alexithymia, depression, attachment styles, and coping strategies of battered women | Journal of Sexual Medicine | 11 | 6 | 1484-1494 | <http://dx.doi.org/10.1111/jsm.12505> | Exclusion reason: No coercive control measure |
| Daly 2008 | Self-reported elder domestic partner violence in one rural iowa county | Journal of Emotional Abuse | 7 | 4 | 115-134 | 10.1300/J135v07n04_06 | Exclusion reason: No coercive control measure |
| deOliveiraFonseca-Machado 2015 | Depressive disorder in pregnant Latin women: Does intimate partner violence matter? | Journal of Clinical Nursing | 24 | 9-10 | 1289-1299 | <http://dx.doi.org/10.1111/jocn.12728> | Exclusion reason: Wrong age group |
| DeMaris 2008 | Partner's stake in conformity and abused wives' psychological trauma | Journal of Interpersonal Violence | 23 | 10 | 1323-1342 | <http://dx.doi.org/10.1177/0886260508314300> | Exclusion reason: Does not report any form of coercive control separately from psychological IPV |
| DePrince 2014 | The geography of intimate partner abuse experiences and clinical responses | Clinical Psychological Science | 2 | 3 | 258-271 | <http://dx.doi.org/10.1177/2167702613507556> | Exclusion reason: No coercive control measure |
| Desmarais 2014 | Intimate partner abuse before and during pregnancy as risk factors for postpartum mental health problems | BMC Pregnancy and Childbirth | 14 | 1 |  | 10.1186/1471-2393-14-132 | Exclusion reason: No coercive control measure |
| Dhairyawan 2013 | Intimate partner violence in women living with HIV attending an inner city clinic in the UK: Prevalence and associated factors | HIV Medicine | 14 | 5 | 303-310 | 10.1111/hiv.12009 | Exclusion reason: No differentiation between types of abuse |
| Dichter 2014 | Associations between psychological, physical, and sexual intimate partner violence and health outcomes among women veteran VA patients | Social Work in Mental Health | 12 | 5-6 | 411-428 | <http://dx.doi.org/10.1080/15332985.2013.870104> | Exclusion reason: No coercive control measure |
| Do 2021 | Intimate partner violence in female same-gender couples: An investigation of actor-partner correlates within the past year | Psychological Trauma:Theory, Pesearch, Practice and Policy | 6 |  | 6 | <https://dx.doi.org/10.1037/tra0001041> | Exclusion reason: No coercive control measure |
| DomenechDelRio 2017 | The Consequences of Intimate Partner Violence on Health: A Further Disaggregation of Psychological Violence-Evidence From Spain | Violence Against Women | 23 | 14 | 1771-1789 | <https://dx.doi.org/10.1177/1077801216671220> | Exclusion reason: Wrong age group |
| dosSantosGomes 2018 | Frailty and life course violence: The international mobility in aging study | Archives of Gerontology and Geriatrics | 76 |  | 26-33 | <http://dx.doi.org/10.1016/j.archger.2018.02.002> | Exclusion reason: No meaningful outcome measures |
| Drouin 2015 | Sexting: A new, digital vehicle for intimate partner aggression? | Computers in Human Behavior | 50 |  | 197-204 | <http://dx.doi.org/10.1016/j.chb.2015.04.001> | Exclusion reason: No coercive control measure |
| Drumm 2009 | Gender variation in partner abuse: Findings from a conservative Christian denomination | Affilia: Journal of Women & Social Work | 24 | 1 | 56-68 | <http://dx.doi.org/10.1177/0886109908326737> | Exclusion reason: No coercive control measure |
| DuMont 2012 | An exploratory study on the consequences and contextual factors of intimate partner violence among immigrant and Canadian-born women | BMJ Open | 2 | 6 |  | <https://dx.doi.org/10.1136/bmjopen-2012-001728> | Exclusion reason: Wrong age group |
| Dutton 2009 | Pathways linking intimate partner violence and posttraumatic disorder | Trauma, Violence, & Abuse | 10 | 3 | 211-224 | <http://dx.doi.org/10.1177/1524838009334451> | Exclusion reason: Wrong study design |
| Dutton 2005 | Patterns of intimate partner violence: Correlates and outcomes | Violence and Victims | 20 | 5 | 483-497 | <http://dx.doi.org/10.1891/vivi.2005.20.5.483> | Exclusion reason: Does not report any form of coercive control separately from psychological IPV |
| Eshelman 2012 | Dating violence: Mental health consequences based on type of abuse | Violence and Victims | 27 | 2 | 215-228 | <http://dx.doi.org/10.1891/0886-6708.27.2.215> | Exclusion reason: No coercive control measure; |
| Esie 2019 | Intimate partner violence and depression in rural Bangladesh: Accounting for violence severity in a high prevalence setting | SSM - Population Health | 7 |  |  | 10.1016/j.ssmph.2019.100368 | Exclusion reason: Wrong age group |
| Estefan 2016 | Depression in women who have left violent relationships: The unique impact of frequent emotional abuse | Violence Against Women | 22 | 11 | 1397-1413 | <http://dx.doi.org/10.1177/1077801215624792> | Exclusion reason: Wrong age group |
| Fisher 2006 | The Extent and Frequency of Abuse in the Lives of Older Women and Their Relationship With Health Outcomes | The Gerontologist | 46 | 2 | 200-209 | <http://dx.doi.org/10.1093/geront/46.2.200> | Exclusion reason: Does not report any form of coercive control separately from psychological IPV |
| Fisher 2013 | Intimate partner violence and perinatal common mental disorders among women in rural Vietnam | International Health | 5 | 1 | 29-37 | <https://dx.doi.org/10.1093/inthealth/ihs012> | Exclusion reason: Does not report any form of coercive control separately from psychological IPV; |
| FitzPatrick 2020 | Physical and Emotional Intimate Partner Violence and Women's Health in the First Year After Childbirth: An Australian Pregnancy Cohort Study | Journal of Interpersonal Violence |  |  | 8.86261E+14 | <https://dx.doi.org/10.1177/0886260520934426> | Exclusion reason: Does not report any form of coercive control separately from psychological IPV; |
| Fleming 2016 | Predicting three types of dissociation in female survivors of intimate partner violence | Journal of Trauma & Dissociation | 17 | 3 | 267-285 | <http://dx.doi.org/10.1080/15299732.2015.1079807> | Exclusion reason: No coercive control measure; |
| Fleming 2016 | Professional versus personal resource utilization in survivors of intimate partner violence | Psychological Trauma: Theory, Research, Practice, and Policy | 8 | 3 | 319-324 | <http://dx.doi.org/10.1037/tra0000074> | Exclusion reason: No meaningful outcome measures |
| Follingstad 2012 | Factors Predicting Relationship Satisfaction, Investment, and Commitment When Women Report High Prevalence of Psychological Abuse | Journal of Family Violence | 27 | 4 | 257-273 | 10.1007/s10896-012-9422-8 | Exclusion reason: No meaningful outcome measures |
| Clements 2005 | Perceived Control and Emotional Status in Abusive College Student Relationships: An Exploration of Gender Differences | Journal of Interpersonal Violence | 20 | 9 | 1058-1077 | <http://dx.doi.org/10.1177/0886260505277939> | Exclusion reason: No differentiation between types of abuse |
| Follingstad 2012 | Women Experiencing Psychological Abuse: Are They a Homogenous Group? | Journal of Aggression, Maltreatment & Trauma | 21 | 8 | 891-916 | 10.1080/10926771.2012.708012 | Exclusion reason: Does not report any form of coercive control separately from psychological IPV |
| Ford-Gilboe 2016 | Development of a brief measure of intimate partner violence experiences: the Composite Abuse Scale (Revised)-Short Form (CASR-SF) | BMJ Open | 6 | 12 | e012824 | <https://dx.doi.org/10.1136/bmjopen-2016-012824> | Exclusion reason: Wrong age group |
| Fortin 2012 | Intimate partner violence and psychological distress among young couples: Analysis of the moderating effect of social support | Journal of Family Violence | 27 | 1 | 63-73 | <http://dx.doi.org/10.1007/s10896-011-9402-4> | Exclusion reason: No coercive control measure |
| Fujiwara 2012 | Factors that contribute to the improvement in maternal parenting after separation from a violent husband or partner | Journal of Interpersonal Violence | 27 | 2 | 380-395 | <http://dx.doi.org/10.1177/0886260511416464> | Exclusion reason: No meaningful outcome measures |
| Fujiwara 2010 | The impact of childhood abuse history and domestic violence on the mental health of women in Japan | Child Abuse & Neglect | 34 | 4 | 267-274 | <http://dx.doi.org/10.1016/j.chiabu.2009.07.007> | Exclusion reason: No differentiation between types of abuse |
| GarcíaOramas 2015 | Mental health in women abused by their partners. A study on samples from Mexico and Spain | Salud Mental | 38 | 5 | 321-327 | 10.17711/SM.0185-3325.2015.044 | Exclusion reason: Wrong language |
| Gervais 2013 | Objectification among college women in the context of intimate partner violence | Violence and Victims | 28 | 1 | 36-49 | <http://dx.doi.org/10.1891/0886-6708.28.1.36> | Exclusion reason: Wrong age group |
| Ghahari 2018 | Marital Conflict, Cognitive Emotion Regulation, Maladaptive Schema and Sexual Satisfaction in spouse abused and non-abused women in Iran: A comparative study | Asian Journal of Psychiatry | 35 |  | 1-2 | <http://dx.doi.org/10.1016/j.ajp.2018.04.012> | Exclusion reason: No differentiation between types of abuse |
| Glenn 2002 | Violence and hostility among families of Vietnam veterans with combat-related posttraumatic stress disorder | Violence and Victims | 17 | 4 | 473-489 | <http://dx.doi.org/10.1891/vivi.17.4.473.33685> | Exclusion reason: No meaningful outcome measures |
| Gobin 2013 | The impact of childhood maltreatment on PTSD symptoms among female survivors of intimate partner violence | Violence and Victims | 28 | 6 | 984-999 | <http://dx.doi.org/10.1891/0886-6708.VV-D-12-00090> | Exclusion reason: No coercive control measure |
| Goessmann 2021 | Toward a Contextually Valid Assessment of Partner Violence: Development and Psycho-Sociometric Evaluation of the Gendered Violence in Partnerships Scale (GVPS) | Frontiers in Psychology | 11 |  |  | 10.3389/fpsyg.2020.607671 | Exclusion reason: Wrong age group |
| Goldstein 2021 | The Effects of Intimate Partner Violence and a History of Childhood Abuse on Mental Health and Stress during Pregnancy | Journal of Family Violence | 36 | 3 | 337-346 | 10.1007/s10896-020-00149-1 | Exclusion reason: No coercive control measure |
| Grande 2003 | Domestic violence in South Australia: a population survey of males and females | Australian & New Zealand Journal of Public Health | 27 | 5 | 543-50 |  | Exclusion reason: No differentiation between types of abuse ; |
| Grandin 1998 | Couple violence and psychological distress | Canadian Journal of Public Health. Revue Canadienne de Sante Publique | 89 | 1 | 43-7 |  | Exclusion reason: No coercive control measure |
| Gulliver 2013 | Exploring risk factors for suicidal ideation in a population-based sample of New Zealand women who have experienced intimate partner violence | Australian & New Zealand Journal of Public Health | 37 | 6 | 527-33 |  | Exclusion reason: Wrong age group |
| Hacialiefendioglu 2021 | Co-occurrence Patterns of Intimate Partner Violence | Pacific Symposium on Biocomputing | 26 |  | 79-90 |  | Exclusion reason: No meaningful outcome measures; |
| Haj-Yahia 2000 | Patterns of violence against engaged Arab women from Israel and some psychological implications | Psychology of Women Quarterly | 24 | 3 | 209-219 | <http://dx.doi.org/10.1111/j.1471-6402.2000.tb00202.x> | Exclusion reason: Wrong age group |
| Hamdan-Mansour 2012 | Evaluating the psychosocial and mental health consequences of abuse among Jordanian women | Eastern Mediterranean Health Journal | 18 | 3 | 205-12 |  | Exclusion reason: Wrong age group |
| Hassan 2012 | Psycho-social correlates of intimate partner violence | Pakistan Journal of Psychological Research | 27 | 2 | 279-295 |  | Exclusion reason: Does not report any form of coercive control separately from psychological IPV |
| Hassanian-Moghaddam 2016 | Violence and Abuse Against Women Who Have Attempted Suicide by Deliberate Self-Poisoning | Journal of Interpersonal Violence | 31 | 7 | 1257-1273 | 10.1177/0886260514564157 | Exclusion reason: Wrong age group |
| Hegarty 2013 | Effect of type and severity of intimate partner violence on women's health and service use: Findings from a primary care trial of women afraid of their partners | Journal of Interpersonal Violence | 28 | 2 | 273-294 | <http://dx.doi.org/10.1177/0886260512454722> | Exclusion reason: Wrong age group |
| Hellemans 2014 | Intimate partner violence in Belgium: Prevalence, individual health outcomes, and relational correlates | Psychologica Belgica | 54 | 1 | 79-96 | <http://dx.doi.org/10.5334/pb.af> | Exclusion reason: Does not report any form of coercive control separately from psychological IPV |
| Hellemans 2015 | Prevalence and impact of Intimate Partner Violence (IPV) among an ethnic minority population | Journal of Interpersonal Violence | 30 | 19 | 3389-3418 | <http://dx.doi.org/10.1177/0886260514563830> | Exclusion reason: Wrong age group |
| Fahmy 2008 | Determinants and health consequences of domestic violence among women in reproductive age at zagazig district, egypt | Journal of the Egyptian Public Health Association | 83 | 1-2 | 87-106 |  | Exclusion reason: No coercive control measure |
| Faisal-Cury 2013 | Temporal relationship between intimate partner violence and postpartum depression in a sample of low income women | Maternal and Child Health Journal | 17 | 7 | 1297-1303 | <http://dx.doi.org/10.1007/s10995-012-1127-3> | Exclusion reason: Wrong age group |
| Fatusi 2006 | Intimate partner violence in Ile-Ife, Nigeria: Women's experiences and men's perspectives | Gender & Behaviour | 4 | 2 | 764-781 |  | Exclusion reason: Unable to obtain full text |
| Fergusson 2005 | Partner violence and mental health outcomes in a New Zealand birth cohort | Journal of Marriage and Family | 67 | 5 | 1103-1119 | <http://dx.doi.org/10.1111/j.1741-3737.2005.00202.x> | Exclusion reason: No coercive control measure |
| Fernandez-Montalvo 2017 | Therapeutic Progression in Abused Women Following a Drug-Addiction Treatment Program | Journal of Interpersonal Violence | 32 | 13 | 2046-2056 | <https://dx.doi.org/10.1177/0886260515591980> | Exclusion reason: No coercive control measure |
| Hines 2010 | A closer look at men who sustain intimate terrorism by women | Partner Abuse | 1 | 3 | 286-313 | <http://dx.doi.org/10.1891/1946-6560.1.3.286> | Exclusion reason: No meaningful outcome measures; |
| Hines 2011 | Understanding the use of violence among men who sustain intimate terrorism | Partner Abuse | 2 | 3 | 259-283 | <http://dx.doi.org/10.1891/1946-6560.2.3.259> | Exclusion reason: Only measures IPV perpetration |
| Hirth 2012 | Racial/ethnic differences in depressive symptoms among young women: The role of intimate partner violence, trauma, and posttraumatic stress disorder | Journal of Women's Health | 21 | 9 | 966-974 | 10.1089/jwh.2011.3366 | Exclusion reason: Wrong age group |
| Honda 2018 | Sexual violence as a key contributor to poor mental health among Japanese women subjected to intimate partner violence | Journal of Women's Health | 27 | 5 | 716-723 | <http://dx.doi.org/10.1089/jwh.2016.6276> | Exclusion reason: Wrong age group |
| Hou 2005 | Domestic violence against women in Taiwan: Their life-threatening situations, post-traumatic responses, and psycho-physiological symptoms. An interview study | International Journal of Nursing Studies | 42 | 6 | 629-636 | <http://dx.doi.org/10.1016/j.ijnurstu.2004.09.011> | Exclusion reason: No differentiation between types of abuse |
| Houry 2006 | Intimate partner violence and mental health symptoms in African American female ED patients | American Journal of Emergency Medicine | 24 | 4 | 444-50 |  | Exclusion reason: No coercive control measure; |
| Houskamp 1991 | The assessment of posttraumatic stress disorder in battered women | Journal of Interpersonal Violence | 6 | 3 | 367-375 | <http://dx.doi.org/10.1177/088626091006003008> | Exclusion reason: No coercive control measure |
| Hussain 2020 | Prevalence and risk factors of domestic violence and its impacts on women’s mental health in Gilgit-Baltistan, Pakistan | Pakistan Journal of Medical Sciences | 36 | 4 | 627-631 | 10.12669/pjms.36.4.1530 | Exclusion reason: No differentiation between types of abuse |
| Huth-Bocks 2013 | Relational trauma and posttraumatic stress symptoms among pregnant women | Psychodynamic Psychiatry | 41 | 2 | 277-301 | <http://dx.doi.org/10.1521/pdps.2013.41.2.277> | Exclusion reason: No coercive control measure; |
| Ilgen 2009 | The association between partner and non-partner aggression and suicidal ideation in patients seeking substance use disorder treatment | Addictive Behaviors | 34 | 2 | 180-186 | <http://dx.doi.org/10.1016/j.addbeh.2008.10.004> | Exclusion reason: Only measures IPV perpetration |
| Ishida 2010 | Exploring the associations between intimate partner violence and women's mental health: Evidence from a population-based study in Paraguay | Social Science & Medicine | 71 | 9 | 1653-1661 | <http://dx.doi.org/10.1016/j.socscimed.2010.08.007> | Exclusion reason: Wrong age group |
| Issahaku 2015 | Health implications of partner violence against women in Ghana | Violence and Victims | 30 | 2 | 250-264 | <http://dx.doi.org/10.1891/0886-6708.VV-D-13-00075> | Exclusion reason: No coercive control measure |
| Iverson 2018 | Adoption, penetration, and effectiveness of a secondary risk screener for intimate partner violence: Evidence to inform screening practices in integrated care settings | General Hospital Psychiatry | 51 |  | 79-84 | 10.1016/j.genhosppsych.2018.01.002 | Exclusion reason: No coercive control measure |
| Iverson 2017 | Intimate Partner Violence Victimization and Associated Implications for Health and Functioning Among Male and Female Post-9/11 Veterans | Medical Care | 55 Suppl 9 Suppl 2 |  | S78-S84 | <https://dx.doi.org/10.1097/MLR.0000000000000741> | Exclusion reason: No coercive control measure |
| Iyengar 2021 | A cross sectional hospital-based study of intimate partner violence and psychiatric comorbidity in pregnancy | Archives of Psychiatry and Psychotherapy | 22 | 4 | 12-21 | 10.12740/APP/120441 | Exclusion reason: No differentiation between types of abuse |
| Jackson 2015 | Intimate partner violence before and during pregnancy: Related demographic and psychosocial factors and postpartum depressive symptoms among Mexican American women | Journal of Interpersonal Violence | 30 | 4 | 659-679 | <http://dx.doi.org/10.1177/0886260514535262> | Exclusion reason: No differentiation between types of abuse |
| Jackson 2020 | Intimate partner violence, firearms, and sleep disturbances: The influence of coercive control and partner firearm ownership | Sleep Health | 6 | 6 | 723-730 | <https://dx.doi.org/10.1016/j.sleh.2020.04.013> | Exclusion reason: No meaningful outcome measures; |
| Jain 2017 | A hospital-based study of intimate partner violence during pregnancy | International Journal of Gynecology and Obstetrics | 137 | 1 | 8-13 | 10.1002/ijgo.12086 | Exclusion reason: No differentiation between types of abuse |
| Jaquier 2013 | Posttraumatic stress and depression symptoms as correlates of deliberate self-harm among community women experiencing intimate partnerviolence | Psychiatry Research | 206 | 1 | 37-42 | 10.1016/j.psychres.2012.09.020 | Exclusion reason: Does not report any form of coercive control separately from psychological IPV |
| Jewkes 2013 | Intimate partner violence as a risk factor for mental health problems in South Africa | Key Issues in Mental Health | 178 |  | 65-74 | 10.1159/000342013 | Exclusion reason: Unable to obtain full text |
| Jina 2012 | Adverse mental health outcomes associated with emotional abuse in young rural South African women: A cross-sectional study | Journal of Interpersonal Violence | 27 | 5 | 862-880 | <http://dx.doi.org/10.1177/0886260511423247> | Exclusion reason: Wrong age group |
| Johnson 2020 | Dyadic correlates of the perpetration of psychological aggression among intimate partners | Psychology of Violence | 10 | 4 | 422-431 | <http://dx.doi.org/10.1037/vio0000257> | Exclusion reason: No coercive control measure |
| Jovanović 2020 | Health consequences of domestic violence against women in Serbia | Vojnosanitetski Pregled | 77 | 1 | 14-21 | 10.2298/VSP171130054M | Exclusion reason: No differentiation between types of abuse |
| Jun 2008 | Intimate partner violence and cigarette smoking: Association between smoking risk and psychological abuse with and without co-occurrence of physical and sexual abuse | American Journal of Public Health | 98 | 3 | 527-535 | <http://dx.doi.org/10.2105/AJPH.2003.037663> | Exclusion reason: No meaningful outcome measures; |
| Kamimura 2014 | Intimate partner violence and physical and mental health among women utilizing community health services in Gujarat, India | BMC Women's Health | 14 |  | 127 | <https://dx.doi.org/10.1186/1472-6874-14-127> | Exclusion reason: No differentiation between types of abuse |
| Kamimura 2016 | Depression and intimate partner violence among college students in Iran | Asian Journal of Psychiatry | 23 |  | 51-55 | <http://dx.doi.org/10.1016/j.ajp.2016.07.014> | Exclusion reason: No coercive control measure |
| Kamimura 2016 | Intimate partner violence-related experiences and mental health among college students in Japan, Singapore, South Korea and Taiwan | International Journal of Social Psychiatry | 62 | 3 | 262-270 | <http://dx.doi.org/10.1177/0020764016629700> | Exclusion reason: No coercive control measure |
| Kandeger 2021 | The mediating effects of self-perception and somatoform dissociation in the relationship between domestic violence and suicidal ideation | Archives of Women's Mental Health | 24 | 2 | 251-257 | <https://dx.doi.org/10.1007/s00737-020-01064-6> | Exclusion reason: No coercive control measure |
| Kanougiya 2021 | Economic abuse and its associations with symptoms of common mental disorders among women in a cross-sectional survey in informal settlements in Mumbai, India | BMC Public Health | 21 | 1 | 842 | <https://dx.doi.org/10.1186/s12889-021-10904-8> | Exclusion reason: No coercive control measure; |
| Lee 2020 | Emotional dysregulation and intimate partner violence: A dyadic perspective | Psychology of Violence | 10 | 2 | 162-171 | <http://dx.doi.org/10.1037/vio0000248> | Exclusion reason: No coercive control measure |
| Leithner 2009 | Physical, sexual, and psychological violence in a gynaecological-psychosomatic outpatient sample: prevalence and implications for mental health | European Journal of Obstetrics, Gynecology, & Reproductive Biology | 144 | 2 | 168-72 | <https://dx.doi.org/10.1016/j.ejogrb.2009.03.003> | Exclusion reason: Violence not (clearly) IPV |
| Lemon 2002 | Preventive healthcare use, smoking, and alcohol use among Rhode Island women experiencing intimate partner violence | Journal of Women's Health & Gender-Based Medicine | 11 | 6 | 555-562 | <http://dx.doi.org/10.1089/152460902760277912> | Exclusion reason: No coercive control measure |
| Lewis 2006 | Coping and Violence Exposure as Predictors of Psychological Functioning in Domestic Violence Survivors | Violence Against Women | 12 | 4 | 340-354 | <http://dx.doi.org/10.1177/1077801206287285> | Exclusion reason: No coercive control measure |
| Lilly 2009 | Ethnicity and risk for symptoms of posttraumatic stress following intimate partner violence: Prevalence and predictors in European American and African American women | Journal of Interpersonal Violence | 24 | 1 | 3-19 | <http://dx.doi.org/10.1177/0886260508314335> | Exclusion reason: No differentiation between types of abuse |
| Longares 2018 | Measuring psychological abuse in same-sex couples: Evidence of validity of the EAPA-P in a Spanish-speaking sample | Anales de Psicologia | 34 | 3 | 555-561 | <http://dx.doi.org/10.6018/analesps.34.3.306281> | Exclusion reason: Does not report any form of coercive control separately from psychological IPV |
| Lowe 2020 | Do levels of posttraumatic growth vary by type of traumatic event experienced? An analysis of the Nurses' Health Study II | Psychological Trauma: Theory, Research, Practice, and Policy |  |  | No Pagination Specified | <http://dx.doi.org/10.1037/tra0000554> | Exclusion reason: No differentiation between types of abuse |
| Ludermir 2008 | Violence against women by their intimate partner and common mental disorders | Social Science & Medicine | 66 | 4 | 1008-1018 | <http://dx.doi.org/10.1016/j.socscimed.2007.10.021> | Exclusion reason: Wrong age group |
| Lysova 2019 | Prevalence and consequences of intimate partner violence in Canada as measured by the national victimization survey | Partner Abuse | 10 | 2 | 199-221 | <http://dx.doi.org/10.1891/1946-6560.10.2.199> | Exclusion reason: Wrong age group |
| MacIsaac 2018 | Prevalence and characteristics of interpersonal violence in people dying from suicide in Victoria, Australia | Asia-Pacific Journal of Public Health | 30 | 1 | 36-44 | <http://dx.doi.org/10.1177/1010539517743615> | Exclusion reason: No coercive control measure |
| Manzolli 2012 | Abuse against women, depression, and infant morbidity: A primary care cohort study in Brazil | American Journal of Preventive Medicine | 43 | 2 | 188-195 | <http://dx.doi.org/10.1016/j.amepre.2012.04.013> | Exclusion reason: No differentiation between types of abuse |
| Martin 2003 | Substance Use Before and During Pregnancy: Links to Intimate Partner Violence | The American Journal of Drug and Alcohol Abuse | 29 | 3 | 599-617 | <http://dx.doi.org/10.1081/ADA-120023461> | Exclusion reason: No coercive control measure |
| Martin 2006 | Intimate Partner Violence and Women's Depression Before and During Pregnancy | Violence Against Women | 12 | 3 | 221-239 | <http://dx.doi.org/10.1177/1077801205285106> | Exclusion reason: No coercive control measure; |
| Maru 2018 | The relationship between intimate partner violence and suicidal ideation among young Chinese, Korean, and Vietnamese American women | Women & Therapy | 41 | 3-4 | 339-355 | <http://dx.doi.org/10.1080/02703149.2018.1430381> | Exclusion reason: No coercive control measure; |
| Massetti 2018 | Healthcare Access and Cancer Screening Among Victims of Intimate Partner Violence | Journal of Women's Health (15409996) | 27 | 5 | 607-614 | 10.1089/jwh.2017.6402 | Exclusion reason: No differentiation between types of abuse |
| Matud 2005 | The psychological impact of domestic violence on Spanish women | Journal of Applied Social Psychology | 35 | 11 | 2310-2322 | <http://dx.doi.org/10.1111/j.1559-1816.2005.tb02104.x> | Exclusion reason: Wrong age group |
| McNamara 2002 | Perceived abuse and disability in a sample of Ohio's women's correctional population | Psychological Reports | 91 | 3,Pt1 | 849-854 | <http://dx.doi.org/10.2466/PR0.91.7.849-854> | Exclusion reason: No differentiation between types of abuse |
| Meekers 2013 | Intimate partner violence and mental health in Bolivia | BMC Women's Health | 13 |  | 28 | <https://dx.doi.org/10.1186/1472-6874-13-28> | Exclusion reason: Wrong age group |
| Mendonca 2017 | Intimate partner violence and incidence of common mental disorder | Revista de Saude Publica | 51 |  | 32 | <https://dx.doi.org/10.1590/S1518-8787.2017051006912> | Exclusion reason: No coercive control measure |
| Mengo 2021 | Intimate Partner Violence and Women's Mental Health: The Mediating Role of Coping Strategies Among Women Seeking Help From the Police | Journal of Interpersonal Violence | 36 | 1/2 | 527-551 | 10.1177/0886260517729402 | Exclusion reason: Violence not (clearly) IPV |
| Montgomery 2015 | Violence against women in selected areas of the United States | American Journal of Public Health | 105 | 10 | 2156-2166 | <http://dx.doi.org/10.2105/AJPH.2014.302430> | Exclusion reason: Violence not (clearly) IPV |
| Moraes 2016 | Intimate partner violence, common mental disorders and household food insecurity: an analysis using path analysis | Public Health Nutrition | 19 | 16 | 2965-2974 |  | Exclusion reason: Wrong age group |
| Morales 2011 | Variables asociadas a abuso físico y psicológico a la pareja | Revista Ciencias de la Salud | 9 | 3 | 271-280 |  | Exclusion reason: Wrong language |
| Morales 2011 | Associated variables with partner physical and psychological abuse | Revista Ciencias de la Salud | 9 | 3 | 271-280 |  | Exclusion reason: Wrong language |
| Katz 1999 | Psychological abuse and depressive symptoms in dating women: Do different types of abuse have differential effects? | Journal of Family Violence | 14 | 3 | 281-295 | <http://dx.doi.org/10.1023/A:1022866400736> | Exclusion reason: Wrong age group |
| Kaufman 2019 | Health and academic consequences of sexual victimisation experiences among students in a university setting | Psychology & Sexuality | 10 | 1 | 56-68 | <http://dx.doi.org/10.1080/19419899.2018.1552184> | Exclusion reason: No coercive control measure; |
| Kayha 2019 | Difficulties in emotion regulation, separation anxiety, and impulsivity as predictors of women's intimate partner violence experiences | Dusunen Adam: Journal of Psychiatry and Neurological Sciences | 32 | 2 | 101-112 |  | Exclusion reason: No differentiation between types of abuse |
| Kelly 2010 | Intimate partner violence, physical health, posttraumatic stress disorder, depression, and quality of life in latinas | The Western Journal of Emergency Medicine | 11 | 3 | 247-51 |  | Exclusion reason: No meaningful outcome measures; |
| Kemp 1995 | Incidence and correlates of posttraumatic stress disorder in battered women: Shelter and community samples | Journal of Interpersonal Violence | 10 | 1 | 43-55 | <http://dx.doi.org/10.1177/088626095010001003> | Exclusion reason: No differentiation between types of abuse |
| Kernic 2003 | Resolution of depression among victims of intimate partner violence: Is cessation of violence enough? | Violence and Victims | 18 | 2 | 115-129 | <http://dx.doi.org/10.1891/vivi.2003.18.2.115> | Exclusion reason: No meaningful outcome measures |
| Khalifeh 2015 | Recent intimate partner violence among people with chronic mental illness: Findings from a national cross-sectional survey | British Journal of Psychiatry | 207 | 3 | 207-212 | 10.1192/bjp.bp.114.144899 | Exclusion reason: Wrong age group |
| Khalkhali 2016 | Domestic Violence in Methamphetamine Psychotic Users, Psychiatric Inpatients, and Healthy People: A Comparative Study | Iranian Journal of Medical Sciences | 41 | 6 | 486-493 |  | Exclusion reason: No meaningful outcome measures; |
| Khan 1993 | MMPI-2 profiles of battered women in transition | Journal of Personality Assessment | 60 | 1 | 100-111 | <http://dx.doi.org/10.1207/s15327752jpa6001_7> | Exclusion reason: No meaningful outcome measures; |
| Khan 2020 | Women's experiences of economic coercion and depressive symptoms in Matlab, Bangladesh | SSM - Population Health | 12 |  | 100641 | <https://dx.doi.org/10.1016/j.ssmph.2020.100641> | Exclusion reason: Wrong age group |
| Kim 2004 | The Association of Antisocial Behavior and Depressive Symptoms Between Partners and Risk for Aggression in Romantic Relationships | Journal of Family Psychology | 18 | 1 | 82-96 | <http://dx.doi.org/10.1037/0893-3200.18.1.82> | Exclusion reason: No coercive control measure |
| Kimerling 2009 | Unemployment among women: Examining the relationship of physical and psychological intimate partner violence and posttraumatic stress disorder | Journal of Interpersonal Violence | 24 | 3 | 450-463 | <http://dx.doi.org/10.1177/0886260508317191> | Exclusion reason: No meaningful outcome measures; |
| Kocot 2003 | The roles of coping and social support in battered women's mental health | Violence Against Women | 9 | 3 | 323-346 | 10.1177/1077801202250075 | Exclusion reason: Does not report any form of coercive control separately from psychological IPV |
| Koopman 2007 | Relationships of Depression to Child and Adult Abuse and Bodily Pain Among Women Who Have Experienced Intimate Partner Violence | Journal of Interpersonal Violence | 22 | 4 | 438-455 | <http://dx.doi.org/10.1177/0886260506297028> | Exclusion reason: Does not report any form of coercive control separately from psychological IPV |
| Kramer 2004 | Prevalence of intimate partner violence and health implications for women using emergency departments and primary care clinics | Womens Health Issues | 14 | 1 | 19-29 |  | Exclusion reason: Violence not (clearly) IPV |
| Kyu 2005 | Prevalence, antecedent causes and consequences of domestic violence in Myanmar | Asian Journal of Social Psychology | 8 | 3 | 244-271 | <http://dx.doi.org/10.1111/j.1467-839X.2005.00170.x> | Exclusion reason: No meaningful outcome measures; |
| LaFlair 2012 | Intimate partner violence/abuse and depressive symptoms among female health care workers: Longitudinal findings | Women's Health Issues | 22 | 1 | e53-e59 | <http://dx.doi.org/10.1016/j.whi.2011.07.001> | Exclusion reason: No differentiation between types of abuse |
| Lacey 2013 | The Impact of Different Types of Intimate Partner Violence on the Mental and Physical Health of Women in Different Ethnic Groups | Journal of Interpersonal Violence | 28 | 2 | 359-385 | 10.1177/0886260512454743 | Exclusion reason: No coercive control measure; |
| Lacey 2021 | Severe Intimate Partner Violence, Sources of Stress and the Mental Health of U.S. Black Women | Journal of Women's Health | 30 | 1 | 17-28 | <https://dx.doi.org/10.1089/jwh.2019.8215> | Exclusion reason: No coercive control measure; |
| Laffaye 2003 | Post-traumatic stress disorder and health-related quality of life in female victims of intimate partner violence | Violence and Victims | 18 | 2 | 227-238 | <http://dx.doi.org/10.1891/vivi.2003.18.2.227> | Exclusion reason: No differentiation between types of abuse |
| Lahav 2019 | Domestic Abuse and Forgiveness among Military Spouses | Journal of Aggression, Maltreatment and Trauma | 28 | 2 | 243-260 | 10.1080/10926771.2018.1531335 | Exclusion reason: No differentiation between types of abuse |
| Lalley-Chareczko 2017 | Sleep disturbance partially mediates the relationship between intimate partner violence and physical/mental health in women and men | Journal of Interpersonal Violence | 32 | 16 | 2471-2495 | <http://dx.doi.org/10.1177/0886260515592651> | Exclusion reason: No coercive control measure; |
| Lambert 2021 | Posttraumatic stress and depression among women in Kenya’s informal settlements: risk and protective factors | European Journal of Psychotraumatology | 12 | 1 |  | 10.1080/20008198.2020.1865671 | Exclusion reason: No coercive control measure; |
| Lamis 2010 | Involvement intimate partner psychological abuse and suicide proneness in college women: Alcohol related problems as a potential mediator | Partner Abuse | 1 | 2 | 169-185 | <http://dx.doi.org/10.1891/1946-6560.1.2.169> | Exclusion reason: Only measures IPV perpetration |
| Lara 2014 | Intimate partner violence and depressive symptoms in pregnant Mexican women: National survey results | Revista de Investigacion Clinica | 66 | 5 | 431-438 |  | Exclusion reason: No differentiation between types of abuse |
| Lee 2019 | Intimate Partner Violence and Psychological Maladjustment: Examining the Role of Institutional Betrayal Among Survivors | Journal of Interpersonal Violence |  |  | 8.86261E+14 | <https://dx.doi.org/10.1177/0886260519836783> | Exclusion reason: Does not report any form of coercive control separately from psychological IPV; |
| Morland 2008 | Intimate partner violence and miscarriage: Examination of the role of physical and psychological abuse and posttraumatic stress disorder | Journal of Interpersonal Violence | 23 | 5 | 652-669 | <http://dx.doi.org/10.1177/0886260507313533> | Exclusion reason: No meaningful outcome measures; |
| Morris 2020 | The Prevalence of Interpersonal Violence (IPV) Against Women and its Associated Variables: >An Exploratory Study in the Rongo Sub-County of Migori County, Kenya | Journal of Interpersonal Violence |  |  | 8.86261E+14 | <https://dx.doi.org/10.1177/0886260520935484> | Exclusion reason: No differentiation between types of abuse |
| Moulding 2020 | Rethinking Women's Mental Health After Intimate Partner Violence | Violence Against Women |  |  | 1077801220921937 | <https://dx.doi.org/10.1177/1077801220921937> | Exclusion reason: No meaningful outcome measures; |
| Mouton 1999 | The associations between health and domestic violence in older women: Results of a pilot study | Journal of Women's Health & Gender-Based Medicine | 8 | 9 | 1173-1179 | <http://dx.doi.org/10.1089/jwh.1.1999.8.1173> | Exclusion reason: Violence not (clearly) IPV |
| Mozzambani 2011 | Psychopathology severity in women victims of violence | Revista de Psiquiatria do Rio Grande do Sul | 33 | 1 | 43-47 | 10.1590/S0101-81082011005000007 | Exclusion reason: Wrong language |
| Mugoya 2020 | Depression and intimate partner violence among urban Kenyan caregivers of children with disabilities | Journal of Psychiatric & Mental Health Nursing | 27 | 1 | 41-53 | <https://dx.doi.org/10.1111/jpm.12550> | Exclusion reason: No coercive control measure; |
| Najavits 2004 | Domestic violence in women with PTSD and substance abuse | Addictive Behaviors | 29 | 4 | 707-715 | <http://dx.doi.org/10.1016/j.addbeh.2004.01.003> | Exclusion reason: No meaningful outcome measures; |
| Nangolo 2003 | Violence against women and its mental health consequences in Namibia | Gender & Behaviour | 1 |  | 16-33 | <http://dx.doi.org/10.4314/gab.v1i1.23310> | Exclusion reason: No meaningful outcome measures; |
| Nathanson 2012 | The prevalence of mental health disorders in a community sample of female victims of intimate partner violence | Partner Abuse | 3 | 1 | 59-75 | <http://dx.doi.org/10.1891/1946-6560.3.1.59> | Exclusion reason: No coercive control measure; |
| Naved 2008 | Spousal violence against women and suicidal ideation in Bangladesh | Women's Health Issues | 18 | 6 | 442-452 | <http://dx.doi.org/10.1016/j.whi.2008.07.003> | Exclusion reason: Wrong age group |
| Nduna 2013 | Prevalence and factors associated with depressive symptoms among young women and men in the Eastern Cape Province, South Africa | Journal of Child and Adolescent Mental Health | 25 | 1 | 43-54 | <http://dx.doi.org/10.2989/17280583.2012.731410> | Exclusion reason: Wrong age group |
| Necho 2020 | The association of intimate partner violence with postpartum depression in women during their first month period of giving delivery in health centers at Dessie town, 2019 | Annals of General Psychiatry | 19 | 1 |  | 10.1186/s12991-020-00310-6 | Exclusion reason: Wrong age group |
| Nelson 1996 | Understanding and treating post-traumatic stress disorder symptoms in female partners of veterans with PTSD | Journal of Marital and Family Therapy | 22 | 4 | 455-467 | <http://dx.doi.org/10.1111/j.1752-0606.1996.tb00220.x> | Exclusion reason: Wrong study design |
| Nhi 2019 | Intimate Partner Violence among Pregnant Women and Postpartum Depression in Vietnam: A Longitudinal Study | BioMed Research International | 2019 |  |  | 10.1155/2019/4717485 | Exclusion reason: Wrong age group |
| Niaz 2002 | Psychological consequences of intimate partner violence: Forms of domestic abuse in both genders | Pakistan Journal of Medical Sciences | 18 | 3 | 205-214 |  | Exclusion reason: No differentiation between types of abuse |
| Nilsson 2008 | Acculturation, partner violence, and psychological distress in refugee women from Somalia | Journal of Interpersonal Violence | 23 | 11 | 1654-1663 | <http://dx.doi.org/10.1177/0886260508314310> | Exclusion reason: No coercive control measure; |
| Nixon 2004 | An exploration of comorbid depression among female victims of intimate partner violence with posttraumatic stress disorder | Journal of Affective Disorders | 82 | 2 | 315-320 | <http://dx.doi.org/10.1016/j.jad.2004.01.008> | Exclusion reason: No coercive control measure; |
| Nurius 2003 | Contextualizing Depression and Physical Functioning in Battered Women: Adding Vulnerability and Resources to the Analysis | Journal of Interpersonal Violence | 18 | 12 | 1411-1431 | <http://dx.doi.org/10.1177/0886260503258033> | Exclusion reason: Does not report any form of coercive control separately from psychological IPV |
| Okafor 2021 | Associations of Emotional, Physical, or Sexual Intimate Partner Violence and Depression Symptoms Among South African Women in a Prospective Cohort Study | Journal of Interpersonal Violence | 36 | 9/10 | NP5060-NP5083 | 10.1177/0886260518796522 | Exclusion reason: No coercive control measure; |
| Orava 1996 | Perceptions of control, depressive symptomatology and self-esteem of women in transition from abusive relationships | Journal of Family Violence | 11 | 2 | 167-186 | <http://dx.doi.org/10.1007/BF02336668> | Exclusion reason: No differentiation between types of abuse |
| Orke 2021 | Attachment Characteristics Among Women Victimized in No, One, and Multiple IPV Relationships: A Case-Control Study | Violence Against Women |  |  | 1077801220981157 | <https://dx.doi.org/10.1177/1077801220981157> | Exclusion reason: No differentiation between types of abuse |
| Peltzer 2017 | Associations between intimate partner violence, depression, and suicidal behavior among women attending antenatal and general outpatients hospital services in Thailand | Nigerian Journal of Clinical Practice | 20 | 7 | 892-899 | <https://dx.doi.org/10.4103/njcp.njcp_453_15> | Exclusion reason: Does not report any form of coercive control separately from psychological IPV |
| Pengpid 2013 | Mental health, partner violence and HIV risk among women with protective orders against violent partners in Vhembe district, South Africa | Asian Journal of Psychiatry | 6 | 6 | 494-499 | <http://dx.doi.org/10.1016/j.ajp.2013.06.005> | Exclusion reason: No meaningful outcome measures; |
| Pengpid 2018 | Intimate partner sexual violence and risk for femicide, suicidality and substance use among women in antenatal care and general out-patients in Thailand | BMC Women's Health | 18 | 1 | 37 | <https://dx.doi.org/10.1186/s12905-018-0526-z> | Exclusion reason: No coercive control measure; |
| Peralta 2003 | Screening for intimate partner violence in a primary care setting: the validity of "feeling safe at home" and prevalence results | Journal of the American Board of Family Practice | 16 | 6 | 525-32 |  | Exclusion reason: No meaningful outcome measures; |
| Perrin 1996 | Assessing the effects of violence on women in battering relationships with the Keane MMPI-PTSD scale | Journal of Traumatic Stress | 9 | 4 | 805-816 | 10.1007/BF02104103 | Exclusion reason: Wrong age group |
| Pico-Alfonso 2006 | The Impact of Physical, Psychological, and Sexual Intimate Male Partner Violence on Women's Mental Health: Depressive Symptoms, Posttraumatic Stress Disorder, State Anxiety, and Suicide | Journal of Women's Health | 15 | 5 | 599-611 | <http://dx.doi.org/10.1089/jwh.2006.15.599> | Exclusion reason: Does not report any form of coercive control separately from psychological IPV |
| Sarasua 2008 | Differential psychopathological profile of victims of intimate partner violence according to age | Psychology in Spain | 12 |  | 53-62 |  | Exclusion reason: No differentiation between types of abuse |
| Scheid 2021 | Feelings and feedings: Psychopathology and breastfeeding attitudes in women with a history of intimate partner violence | Psychological Trauma: Theory, Research, Practice, and Policy | 13 | 3 | 394-402 | <http://dx.doi.org/10.1037/tra0000984> | Exclusion reason: No differentiation between types of abuse |
| Mills 2018 | Mediated effects of coping on mental health outcomes of African American women exposed to physical and psychological abuse | Violence Against Women | 24 | 2 | 186-206 | <http://dx.doi.org/10.1177/1077801216686219> | Exclusion reason: No coercive control measure; |
| Miszkurka 2012 | Immigrant status, antenatal depressive symptoms, and frequency and source of violence: what's the relationship? | Archives of Women's Mental Health | 15 | 5 | 387-396 | 10.1007/s00737-012-0298-7 | Exclusion reason: No differentiation between types of abuse |
| Schrag 2015 | Economic abuse and later material hardship: Is depression a mediator? | Affilia: Journal of Women & Social Work | 30 | 3 | 341-351 | <http://dx.doi.org/10.1177/0886109914541118> | Exclusion reason: No coercive control measure; |
| Schraiber 2010 | Validity of the WHO VAW study instrument for estimating gender-based violence against women | Revista de Saude Publica | 44 | 4 | 658-66 |  | Exclusion reason: Wrong language |
| Scrafford 2019 | Effects of intimate partner violence, mental health, and relational resilience on perinatal health | Journal of Traumatic Stress | 32 | 4 | 506-515 | <http://dx.doi.org/10.1002/jts.22414> | Exclusion reason: No differentiation between types of abuse |
| Sediri 2020 | Women's mental health: acute impact of COVID-19 pandemic on domestic violence | Archives of Women's Mental Health | 23 | 6 | 749-756 | <https://dx.doi.org/10.1007/s00737-020-01082-4> | Exclusion reason: No differentiation between types of abuse |
| Seedat 2005 | Association Between Physical Partner Violence, Posttraumatic Stress, Childhood Trauma, and Suicide Attempts in a Community Sample of Women | Violence and Victims | 20 | 1 | 87-98 | <http://dx.doi.org/10.1891/vivi.2005.20.1.87> | Exclusion reason: No coercive control measure; |
| Sezgin 2019 | Type of Traumatic Events, Mental Health Problems, and Posttraumatic Cognitions Among Eastern Anatolian Women | Journal of Interpersonal Violence |  |  | 8.86261E+14 | <https://dx.doi.org/10.1177/0886260519858385> | Exclusion reason: Wrong age group |
| Shah 2018 | Intimate partner violence and psychotic experiences in four U.S. cities | Schizophrenia Research | 195 |  | 506-512 | 10.1016/j.schres.2017.09.017 | Exclusion reason: No coercive control measure; |
| Shamu 2016 | High-frequency intimate partner violence during pregnancy, postnatal depression and suicidal tendencies in Harare, Zimbabwe | General Hospital Psychiatry | 38 |  | 109-114 | 10.1016/j.genhosppsych.2015.10.005 | Exclusion reason: Wrong age group |
| Shannon 2008 | An examination of women's alcohol use and partner victimization experiences among women with protective orders | Substance Use & Misuse | 43 | 8-9 | 1110-1128 | <http://dx.doi.org/10.1080/10826080801918155> | Exclusion reason: Wrong age group |
| Shannon 2016 | Examining intimate partner violence and health factors among rural Appalachian pregnant women | Journal of Interpersonal Violence | 31 | 15 | 2622-2640 | <http://dx.doi.org/10.1177/0886260515579508> | Exclusion reason: No meaningful outcome measures; |
| Sheikhan 2014 | Domestic violence in Iranian infertile women | Medical Journal of the Islamic Republic of Iran | 28 |  | 152 |  | Exclusion reason: No differentiation between types of abuse |
| Shen 2019 | Intimate partner violence and psychological distress among emerging adult women: A bidirectional relationship | Journal of Women's Health | 28 | 8 | 1060-1067 | <http://dx.doi.org/10.1089/jwh.2018.7405> | Exclusion reason: No coercive control measure; |
| Shepherd-McMullen 2015 | Negative mood regulation expectancies moderate the relationship between psychological abuse and avoidant coping | Journal of Interpersonal Violence | 30 | 9 | 1553-1566 | <http://dx.doi.org/10.1177/0886260514540805> | Exclusion reason: No meaningful outcome measures; |
| Shevlin 2013 | Patterns of lifetime female victimisation and psychotic experiences: A study based on the UK Adult Psychiatric Morbidity Survey 2007 | Social Psychiatry and Psychiatric Epidemiology: The International Journal for Research in Social and Genetic Epidemiology and Mental Health Services | 48 | 1 | 15-24 | <http://dx.doi.org/10.1007/s00127-012-0573-y> | Exclusion reason: Wrong age group |
| Shorey 2017 | Examining the reactions of women in substance use treatment as participants in a study on intimate partner violence: Does shame proneness matter | Partner Abuse | 8 | 4 | 395-408 | <http://dx.doi.org/10.1891/1946-6560.8.4.395> | Exclusion reason: No coercive control measure; |
| Signorelli 2020 | Depression, PTSD and alexithymia in victims of intimate partner violence: a case-control study | Revista de Psiquiatria Clinica | 47 | 2 | 45-50 | 10.1590/0101-60830000000230 | Exclusion reason: No coercive control measure; |
| Silva 2015 | Health-related factors associated with intimate partner violence in women attending a primary care clinic in south-western Nigeria | South African Family Practice | 57 | 2 | 69-76 | 10.1080/20786190.2014.976994 | Exclusion reason: Wrong age group |
| Skomorovsky 2006 | The buffering role of social support perceptions in relation to eating disturbances among women in abusive dating relationships | Sex Roles: A Journal of Research | 54 | 9-10 | 627-638 | <http://dx.doi.org/10.1007/s11199-006-9030-2> | Exclusion reason: No coercive control measure; |
| Smith 2013 | The Self Assessment of Future Events Scale (SAFE): Assessing perceptions of risk for future violence in intimate partner relationships | Journal of Marital and Family Therapy | 39 | 3 | 314-329 | <http://dx.doi.org/10.1111/j.1752-0606.2012.00319.x> | Exclusion reason: No coercive control measure; |
| Soleimani 2017 | Health consequences of intimate partner violence against married women: A population-based study in northern Iran | Psychology, Health & Medicine | 22 | 7 | 845-850 | <http://dx.doi.org/10.1080/13548506.2016.1263755> | Exclusion reason: No coercive control measure; |
| Solinas-Saunders 2021 | Perpetration and Victimization of Emotional Abuse and Controlling Behaviors in a Sample of Batterer Intervention Program’s Participants: An Analysis of Stressors and Risk Factors | Crime and Delinquency |  |  |  | 10.1177/0011128721999349 | Exclusion reason: Wrong age group |
| Sorbo 2014 | Adult physical, sexual, and emotional abuse and postpartum depression, a population based, prospective study of 53,065 women in the Norwegian Mother and Child Cohort Study | BMC Pregnancy & Childbirth | 14 |  | 316 | <https://dx.doi.org/10.1186/1471-2393-14-316> | Exclusion reason: Violence not (clearly) IPV |
| Stein 2021 | Intimate Partner Violence Among Surgeons: We are Not Immune | Annals of Surgery | 273 | 3 | 387-392 | <https://dx.doi.org/10.1097/SLA.0000000000004553> | Exclusion reason: No differentiation between types of abuse |
| Stene 2010 | Psychotropic drug use among women exposed to intimate partner violence: A population-based study | Scandinavian Journal of Public Health | 38 | Suppl 5 | 88-95 | <http://dx.doi.org/10.1177/1403494810382815> | Exclusion reason: No coercive control measure; |
| Stoliker 2018 | An examination of the effects of different victimization types on psychological and behavioral health outcomes and the mediating role of stress | Victims & Offenders | 13 | 6 | 834-858 | <http://dx.doi.org/10.1080/15564886.2018.1491436> | Exclusion reason: Wrong age group |
| Straight 2003 | The impact of partner psychological abuse on health behaviors and health status in college women | Journal of Interpersonal Violence | 18 | 9 | 1035-1054 | <http://dx.doi.org/10.1177/0886260503254512> | Exclusion reason: Wrong age group |
| Straus 2009 | Intimate partner violence and functional health status: Associations with severity, danger, and self-advocacy behaviors | Journal of Women's Health | 18 | 5 | 625-631 | <http://dx.doi.org/10.1089/jwh.2007.0521> | Exclusion reason: Duplicate publication |
| Straus 2009 | Intimate partner violence and functional health status: associations with severity, danger, and self-advocacy behaviors [corrected] [published erratum appears in J WOMENS HEALTH 2009 Jun;18(6):917] | Journal of Women's Health (15409996) | 18 | 5 | 625-631 | 10.1089/jwh.2007.0521 | Exclusion reason: No coercive control measure; |
| Tanimu 2016 | The pattern and correlates of intimate partner violence among women in Kano, Nigeria | African Journal of Primary Health Care & Family Medicine | 8 | 1 | e1-e6 | <https://dx.doi.org/10.4102/phcfm.v8i1.1209> | Exclusion reason: Wrong age group |
| Tasa-Vinyals 2020 | Intimate Partner Violence Among Patients Diagnosed With Severe Mental Disorder | Journal of Nervous & Mental Disease | 208 | 10 | 749-754 | <https://dx.doi.org/10.1097/NMD.0000000000001207> | Exclusion reason: No differentiation between types of abuse |
| Tavoli 2016 | Quality of life in women who were exposed to domestic violence during pregnancy | BMC Pregnancy & Childbirth | 16 |  | 19 | <https://dx.doi.org/10.1186/s12884-016-0810-6> | Exclusion reason: Wrong age group |
| Polychronopoulou 2016 | The psychosocial repercussions of domestic violence in battered women | Psychiatriki | 27 | 2 | 148-9 |  | Exclusion reason: Wrong language |
| Porcerelli 2006 | Physical and psychological symptoms in emotionally abused and non-abused women | Journal of the American Board of Family Medicine: JABFM | 19 | 2 | 201-4 |  | Exclusion reason: Violence not (clearly) IPV |
| Porrua-Garcia 2016 | Development and validation ofthe scale of psychological abuse in intimate partner violence (EAPA-P) | Psicothema | 28 | 2 | 214-221 |  | Exclusion reason: Does not report any form of coercive control separately from psychological IPV |
| Potter 2020 | Categories and health impacts of intimate partner violence in the World Health Organization multi-country study on women's health and domestic violence | International Journal of Epidemiology | 12 |  | 12 | <https://dx.doi.org/10.1093/ije/dyaa220> | Exclusion reason: Wrong age group |
| Prasad 2018 | Intimate partner violence: factors and types of abuse women face in and around Coimbatore District, Tamilnadu | Indian Journal of Public Health Research and Development | 9 | 11 | 67-70 | 10.5958/0976-5506.2018.01427.4 | Exclusion reason: Wrong study design |
| Price 2019 | Experiences of Reproductive Coercion in Queensland Women | Journal of Interpersonal Violence |  |  | 8.86261E+14 | <https://dx.doi.org/10.1177/0886260519846851> | Exclusion reason: Wrong age group |
| Prospero 2010 | Sexual coercion and mental health symptoms among heterosexual men: The pressure to say "yes" | American Journal of Men's Health | 4 | 2 | 98-103 | <http://dx.doi.org/10.1177/1557988308330106> | Exclusion reason: No coercive control measure; |
| Raffo 2010 | Psychological and physical abuse among pregnant women in a Medicaid-sponsored prenatal program | Public Health Nursing | 27 | 5 | 385-398 | <http://dx.doi.org/10.1111/j.1525-1446.2010.00871.x> | Exclusion reason: Violence not (clearly) IPV |
| Ramiro 2004 | Risk markers of severe psychological violence against women: a WorldSAFE multi-country study | Injury Control & Safety Promotion | 11 | 2 | 131-7 |  | Exclusion reason: Wrong age group |
| Ratner 1993 | The incidence of wife abuse and mental health status in abused wives in Edmonton, Alberta | Canadian Journal of Public Health. Revue Canadienne de Sante Publique | 84 | 4 | 246-9 |  | Exclusion reason: No coercive control measure; |
| Rauer 2010 | Sleeping with one eye open: Marital abuse as an antecedent of poor sleep | Journal of Family Psychology | 24 | 6 | 667-677 | <http://dx.doi.org/10.1037/a0021354> | Exclusion reason: No differentiation between types of abuse ; |
| Records 2005 | A comparative study of postpartum depression in abused and nonabused women | Archives of Psychiatric Nursing | 19 | 6 | 281-290 | <http://dx.doi.org/10.1016/j.apnu.2005.07.010> | Exclusion reason: No differentiation between types of abuse ; |
| Reddy 2020 | Childhood abuse and intimate partner violence among women with mood disorders | Journal of Affective Disorders | 272 |  | 335-339 | <http://dx.doi.org/10.1016/j.jad.2020.03.113> | Exclusion reason: No meaningful outcome measures |
| Rees 2016 | A high-risk group of pregnant women with elevated levels of conflict-related trauma, intimate partner violence, symptoms of depression and other forms of mental distress in post-conflict Timor-Leste | Transl Psychiatry Psychiatry | 6 |  | e725 | <https://dx.doi.org/10.1038/tp.2015.212> | Exclusion reason: Does not report any form of coercive control separately from psychological IPV |
| Richardson 2020 | The effect of intimate partner violence on women's mental distress: a prospective cohort study of 3010 rural Indian women | Social Psychiatry & Psychiatric Epidemiology | 55 | 1 | 71-79 | <https://dx.doi.org/10.1007/s00127-019-01735-5> | Exclusion reason: Wrong age group |
| Rogers 2014 | Women's exposure to psychological abuse: Does that experience predict mental health outcomes? | Journal of Family Violence | 29 | 6 | 595-611 | <http://dx.doi.org/10.1007/s10896-014-9621-6> | Exclusion reason: Does not report any form of coercive control separately from psychological IPV |
| Roh 2016 | Risk and protective factors for depressive symptoms among indigenous older adults: Intimate partner violence (IPV) and social support | Journal of Gerontological Social Work | 59 | 4 | 316-331 | <http://dx.doi.org/10.1080/01634372.2016.1214659> | Exclusion reason: No coercive control measure; |
| Romito 2005 | The impact of current and past interpersonal violence on women's mental health | Social Science & Medicine | 60 | 8 | 1717-1727 | <http://dx.doi.org/10.1016/j.socscimed.2004.08.026> | Exclusion reason: Wrong age group |
| Rosen 2002 | Gender differences in the experience of intimate partner violence among active duty U.S. Army soldiers | Military Medicine | 167 | 12 | 959-963 |  | Exclusion reason: No coercive control measure; |
| Ruiz-Perez 2005 | Intimate Partner Violence and Mental Health Consequences in Women Attending Family Practice in Spain | Psychosomatic Medicine | 67 | 5 | 791-797 | <http://dx.doi.org/10.1097/01.psy.0000181269.11979.cd> | Exclusion reason: No coercive control measure; |
| Ruiz-Pérez 2018 | Intimate partner violence and mental disorders: Co-occurrence and gender differences in a large cross-sectional population based study in Spain | Journal of Affective Disorders | 229 |  | 69-78 | 10.1016/j.jad.2017.12.032 | Exclusion reason: No coercive control measure; |
| Rurangirwa 2018 | Intimate partner violence during pregnancy in relation to non-psychotic mental health disorders in Rwanda: A cross-sectional population-based study | BMJ Open | 8 | 7 |  | 10.1136/bmjopen-2018-021807 | Exclusion reason: Wrong age group |
| Sabina 2017 | Problematic alcohol and drug use and the risk of partner violence victimization among male and female college students | Journal of Family Violence | 32 | 3 | 305-316 | <http://dx.doi.org/10.1007/s10896-017-9907-6> | Exclusion reason: No coercive control measure; |
| Sabina 2008 | Polyvictimization by dating partners and mental health among U.S. college students | Violence and Victims | 23 | 6 | 667-682 | <http://dx.doi.org/10.1891/0886-6708.23.6.667> | Exclusion reason: No coercive control measure; |
| Sabri 2013 | Victimization experiences, substance misuse, and mental health problems in relation to risk for lethality among African American and African Caribbean women | Journal of Interpersonal Violence | 28 | 16 | 3223-41 | <https://dx.doi.org/10.1177/0886260513496902> | Exclusion reason: Does not report any form of coercive control separately from psychological IPV |
| Saito 2012 | Effect of intimate partner violence on postpartum women's health in northeastern Thailand | Nursing & Health Sciences | 14 | 3 | 345-351 | <http://dx.doi.org/10.1111/j.1442-2018.2012.00735.x> | Exclusion reason: Does not report any form of coercive control separately from psychological IPV |
| Saito 2013 | Effect of intimate partner violence on antenatal functional health status of childbearing women in Northeastern Thailand | Health Care for Women International | 34 | 9 | 757-774 | <http://dx.doi.org/10.1080/07399332.2013.794459> | Exclusion reason: Duplicate publication |
| Salcioglu 2017 | Anticipatory fear and helplessness predict PTSD and depression in domestic violence survivors | Psychological Trauma: Theory, Research, Practice, and Policy | 9 | 1 | 117-125 | <http://dx.doi.org/10.1037/tra0000200> | Exclusion reason: Violence not (clearly) IPV; |
| Salom 2015 | Substance use and mental health disorders are linked to different forms of intimate partner violence victimisation | Drug and Alcohol Dependence | 151 |  | 121-127 | 10.1016/j.drugalcdep.2015.03.011 | Exclusion reason: Does not report any form of coercive control separately from psychological IPV |
| Salwen 2015 | Sexual coercion and psychological aggression victimization: Unique constructs and predictors of depression | Partner Abuse | 6 | 4 | 367-382 | <http://dx.doi.org/10.1891/1946-6560.6.4.367> | Exclusion reason: No coercive control measure; |
| Samelius 2010 | Lifetime history of abuse, suffering and psychological health | Nordic Journal of Psychiatry | 64 | 4 | 227-232 | <http://dx.doi.org/10.3109/08039480903478680> | Exclusion reason: Violence not (clearly) IPV; |
| Samios 2020 | Meaning in Life Following Intimate Partner Psychological Aggression: The Roles of Self-Kindness, Positive Reframing, and Growth | Journal of Interpersonal Violence | 35 | 7/8 | 1567-1586 | 10.1177/0886260519898437 | Exclusion reason: Wrong age group |
| Santos 2018 | Domains of common mental disorders in women reporting intimate partner violence | Revista Latino-Americana de Enfermagem | 26 |  | e3099 | <https://dx.doi.org/10.1590/1518-8345.2740.3099> | Exclusion reason: No coercive control measure; |
| Terrazas-Carrillo 2016 | Depression among Mexican women: The impact of nonviolent coercive control, intimate partner violence and employment status | Journal of Family Violence | 31 | 6 | 721-734 | <http://dx.doi.org/10.1007/s10896-016-9827-x> | Exclusion reason: Wrong age group |
| Theran 2006 | Abusive Partners and Ex-Partners: Understanding the Effects of Relationship to the Abuser on Women's Well-Being | Violence Against Women | 12 | 10 | 950-969 | <http://dx.doi.org/10.1177/1077801206292871> | Exclusion reason: Wrong age group |
| ThoTran 2018 | Emotional violence exerted by intimate partners and postnatal depressive symptoms among women in Vietnam: A prospective cohort study | PLoS ONE [Electronic Resource] | 13 | 11 |  | 10.1371/journal.pone.0207108 | Exclusion reason: No coercive control measure; |
| Tiwari 2008 | The impact of psychological abuse by an intimate partner on the mental health of pregnant women | BJOG: An International Journal of Obstetrics & Gynaecology | 115 | 3 | 377-84 | <https://dx.doi.org/10.1111/j.1471-0528.2007.01593.x> | Exclusion reason: No coercive control measure; |
| Tiwari 2013 | Factors mediating the relationship between intimate partner violence and chronic pain in Chinese women | Journal of Interpersonal Violence | 28 | 5 | 1067-1087 | <http://dx.doi.org/10.1177/0886260512459380> | Exclusion reason: No coercive control measure; |
| Tran 2019 | Are peritraumatic perceptions of fear/life threat and posttraumatic negative self-conscious appraisals/emotions differentially associated with PTSD symptoms? | Cognitive Therapy and Research | 43 | 1 | 272-283 | <http://dx.doi.org/10.1007/s10608-018-9903-z> | Exclusion reason: No differentiation between types of abuse |
| Trimpey 1989 | Self-esteem and anxiety: key issues in an abused women's support group | Issues in Mental Health Nursing | 10 | 3-4 | 297-308 |  | Exclusion reason: No differentiation between types of abuse |
| Tuel 1998 | Self-esteem and depression in battered women: A comparison of lesbian and heterosexual survivors | Violence Against Women | 4 | 3 | 344-362 | <http://dx.doi.org/10.1177/1077801298004003006> | Exclusion reason: No coercive control measure; |
| Tuten 2004 | Partner violence impacts the psychosocial and psychiatric status of pregnant, drug-dependent women | Addictive Behaviors | 29 | 5 | 1029-1034 | 10.1016/j.addbeh.2004.02.055 | Exclusion reason: No differentiation between types of abuse ; |
| Tyson 2007 | Beyond violence: Threat reappraisal in women recently separated from intimate-partner violent relationships | Journal of Social and Personal Relationships | 24 | 5 | 693-706 | <http://dx.doi.org/10.1177/0265407507081455> | Exclusion reason: Does not report any form of coercive control separately from psychological IPV; |
| Urquia 2011 | Experiences of violence before and during pregnancy and adverse pregnancy outcomes: an analysis of the Canadian Maternity Experiences Survey | BMC Pregnancy & Childbirth | 11 | 1 | 42-42 | 10.1186/1471-2393-11-42 | Exclusion reason: Wrong age group |
| Varma 2007 | Intimate partner violence and sexual coercion among pregnant women in India: Relationship with depression and post-traumatic stress disorder | Journal of Affective Disorders | 102 | 1-3 | 227-235 | <http://dx.doi.org/10.1016/j.jad.2006.09.026> | Exclusion reason: Does not report any form of coercive control separately from psychological IPV; |
| Veloso 2019 | CONSUMPTION OF ALCOHOL AND TOBACCO BY WOMEN AND THE OCCURRENCE OF VIOLENCE BY INTIMATE PARTNER | Texto & Contexto Enfermagem | 28 |  | 1-17 | 10.1590/1980-265X-TCE-2017-0581 | Exclusion reason: No differentiation between types of abuse |
| Vidourek 2017 | Emotional abuse: Correlates to abuse among college students | Journal of Aggression, Maltreatment & Trauma | 26 | 7 | 792-803 | <http://dx.doi.org/10.1080/10926771.2017.1308980> | Exclusion reason: Violence not (clearly) IPV; |
| Vilarino 2018 | Psychological harm in women victims of intimate partner violence: Epidemiology and quantification of injury in mental health markers | Psychosocial Intervention | 27 | 3 | 145-152 | <http://dx.doi.org/10.5093/pi2018a23> | Exclusion reason: No differentiation between types of abuse |
| Vitanza 1995 | Distress and symptoms of posttraumatic stress disorder in abused women | Violence and Victims | 10 | 1 | 23-34 | <http://dx.doi.org/10.1891/0886-6708.10.1.23> | Exclusion reason: No differentiation between types of abuse |
| Vizcarra 2004 | Partner violence as a risk factor for mental health among women from communities in the Philippines, Egypt, Chile, and India | Injury Control & Safety Promotion | 11 | 2 | 125-9 |  | Exclusion reason: Wrong age group |
| Vogel 2001 | PTSD symptoms and partner abuse: Low income women at risk | Journal of Traumatic Stress | 14 | 3 | 569-584 | 10.1023/A:1011116824613 | Exclusion reason: No differentiation between types of abuse |
| Watlington 2006 | The Roles of Religion and Spirituality Among African American Survivors of Domestic Violence | Journal of Clinical Psychology | 62 | 7 | 837-857 | <http://dx.doi.org/10.1002/jclp.20268> | Exclusion reason: No coercive control measure |
| Weaver 2020 | Intimate Partner Violence and Body Shame: An Examination of the Associations Between Abuse Components and Body-Focused Processes | Violence Against Women | 26 | 12-13 | 1538-1554 | <https://dx.doi.org/10.1177/1077801219873434> | Exclusion reason: Does not report any form of coercive control separately from psychological IPV; |
| Weaver 2020 | The Relationship Between Women’s Resources and Health-Related Quality of Life in a Sample of Female Victims of Intimate Partner Violence | Journal of Social Service Research |  |  |  | 10.1080/01488376.2020.1859433 | Exclusion reason: Does not report any form of coercive control separately from psychological IPV; |
| Webermann 2021 | Intimate Partner Violence Among Patients With Dissociative Disorders | Journal of Interpersonal Violence | 36 | 3/4 | NP1441-1462NP | 10.1177/0886260517746943 | Exclusion reason: No differentiation between types of abuse |
| Wei 2019 | Prevalence of Intimate Partner Violence and Associated Factors Among Men Who Have Sex with Men in China | Journal of Interpersonal Violence |  |  | 8.86261E+14 | <https://dx.doi.org/10.1177/0886260519889935> | Exclusion reason: No meaningful outcome measures |
| Weiss 2015 | The underlying role of posttraumatic stress disorder symptoms in the association between intimate partner violence and deliberate self-harm among African American women | Comprehensive Psychiatry | 59 |  | 8-16 | 10.1016/j.comppsych.2014.05.018 | Exclusion reason: No coercive control measure; |
| Wijma 2007 | The association between ill-health and abuse: a cross-sectional population based study | Scandinavian Journal of Psychology | 48 | 6 | 567-75 |  | Exclusion reason: Violence not (clearly) IPV; |
| Willie 2018 | The Impact of Adverse Childhood Events on the Sexual and Mental Health of Women Experiencing Intimate Partner Violence | Journal of Interpersonal Violence |  |  | 8.86261E+14 | <https://dx.doi.org/10.1177/0886260518802852> | Exclusion reason: No meaningful outcome measures |
| Wolford-Clevenger 2016 | Dating violence victimization, interpersonal needs, and suicidal ideation among college students | Crisis: The Journal of Crisis Intervention and Suicide Prevention | 37 | 1 | 51-58 | <http://dx.doi.org/10.1027/0227-5910/a000353> | Exclusion reason: No coercive control measure; |
| Wolford-Clevenger 2016 | The association of partner abuse types and suicidal ideation among men and women college students | Violence and Victims | 31 | 3 | 471-485 | <http://dx.doi.org/10.1891/0886-6708.VV-D-14-00083> | Exclusion reason: Wrong age group |
| Wong 2016 | Problem-focused coping mediates the impact of intimate partner violence on mental health among Chinese women | Psychology of Violence | 6 | 2 | 313-322 | <http://dx.doi.org/10.1037/a0039496> | Exclusion reason: No differentiation between types of abuse |
| Wong 2011 | Depression among women experiencing intimate partner violence in a Chinese community | Nursing Research | 60 | 1 | 58-65 | <http://dx.doi.org/10.1097/NNR.0b013e3182002a7c> | Exclusion reason: No coercive control measure; |
| Woods 2000 | Prevalence and patterns of posttraumatic stress disorder in abused and postabused women | Issues in Mental Health Nursing | 21 | 3 | 309-324 | <http://dx.doi.org/10.1080/016128400248112> | Exclusion reason: No coercive control measure; |
| Woods 2008 | Physical health and posttraumatic stress disorder symptoms in women experiencing intimate partner violence | Journal of Midwifery & Women's Health | 53 | 6 | 538-546 | 10.1016/j.jmwh.2008.07.004 | Exclusion reason: Wrong age group; |
| Woolhouse 2012 | Depressive symptoms and intimate partner violence in the 12 months after childbirth: a prospective pregnancy cohort study | BJOG: An International Journal of Obstetrics & Gynaecology | 119 | 3 | 315-23 | <https://dx.doi.org/10.1111/j.1471-0528.2011.03219.x> | Exclusion reason: Does not report any form of coercive control separately from psychological IPV |
| Sullivan 2009 | Testing posttraumatic stress as a mediator of physical, sexual, and psychological intimate partner violence and substance problems among women | Journal of Traumatic Stress | 22 | 6 | 575-584 |  | Exclusion reason: Does not report any form of coercive control separately from psychological IPV |
| Sullivan 2017 | Is firearm threat in intimate relationships associated with posttraumatic stress disorder symptoms among women? | Violence and Gender | 4 | 2 | 31-36 | <http://dx.doi.org/10.1089/vio.2016.0024> | Exclusion reason: Does not report any form of coercive control separately from psychological IPV; |
| Sumner 2011 | The influence of prenatal trauma, stress, social support, and years of residency in the US on postpartum maternal health status among low-income Latinas | Maternal and Child Health Journal | 15 | 7 | 1046-1054 | <http://dx.doi.org/10.1007/s10995-010-0649-9> | Exclusion reason: No differentiation between types of abuse |
| Sussex 2005 | The Impact of Domestic Violence on Depression in Teen Mothers: Is the Fear or Threat of Violence Sufficient? | Brief Treatment and Crisis Intervention | 5 | 1 | 109-120 | <http://dx.doi.org/10.1093/brief-treatment/mhi005> | Exclusion reason: No differentiation between types of abuse |
| Sutherland 1998 | The long-term effects of battering on women's health | Womens Health | 4 | 1 | 41-70 |  | Exclusion reason: No differentiation between types of abuse |
| Swan 2003 | Behavioral and psychological differences among abused women who use violence in intimate relationships | Violence Against Women | 9 | 1 | 75-109 | <http://dx.doi.org/10.1177/1077801202238431> | Exclusion reason: No meaningful outcome measures |
| Tadegge 2008 | The mental health consequences of intimate partner violence against women in Agaro Town, southwest Ethiopia | Tropical Doctor | 38 | 4 | 228-229 | 10.1258/td.2008.070353 | Exclusion reason: Wrong age group; |
| Taft 2009 | Posttraumatic stress disorder symptoms, relationship adjustment, and relationship aggression in a sample of female flood victims | Journal of Family Violence | 24 | 6 | 389-396 | 10.1007/s10896-009-9241-8 | Exclusion reason: No coercive control measure; |
| Taft 2006 | Examining the correlates of psychological aggression among a community sample of couples | Journal of Family Psychology | 20 | 4 | 581-588 | <http://dx.doi.org/10.1037/0893-3200.20.4.581> | Exclusion reason: Does not report any form of coercive control separately from psychological IPV; |
| Taft 2007 | Examining the correlates of engagement and disengagement coping among help-seeking battered women | Violence and Victims | 22 | 1 | 3-17 | <http://dx.doi.org/10.1891/vv-v22i1a001> | Exclusion reason: No meaningful outcome measures |
| Taft 2007 | Posttraumatic stress disorder and physical health symptoms among women seeking help for relationship aggression | Journal of Family Psychology | 21 | 3 | 354-362 | <http://dx.doi.org/10.1037/0893-3200.21.3.354> | Exclusion reason: Duplicate publication; |
| Yanqiu 2011 | Suicidal ideation and the prevalence of intimate partner violence against women in rural Western China | Violence Against Women | 17 | 10 | 1299-1312 | <http://dx.doi.org/10.1177/1077801211425217> | Exclusion reason: Wrong age group; |
| Yoshihama 2009 | The role of emotional abuse in intimate partner violence and health among women in Yokohama, Japan | American Journal of Public Health | 99 | 4 | 647-653 | 10.2105/AJPH.2007.118976 | Exclusion reason: Does not report any form of coercive control separately from psychological IPV |
| Yu 2018 | Association of intimate partner violence during pregnancy, prenatal depression, and adverse birth outcomes in Wuhan, China | BMC Pregnancy & Childbirth | 18 | 1 | 469 | <https://dx.doi.org/10.1186/s12884-018-2113-6> | Exclusion reason: No coercive control measure; |
| Yuan 2019 | Intimate Partner Violence and Depression in Women in China | Journal of Interpersonal Violence |  |  | 8.86261E+14 | <https://dx.doi.org/10.1177/0886260519888538> | Exclusion reason: Does not report any form of coercive control separately from psychological IPV; |
| Zacarias 2012 | Symptoms of depression, anxiety, and somatization in female victims and perpetrators of intimate partner violence in Maputo City, Mozambique | International Journal of Women's Health | 4 |  | 491-503 | <https://dx.doi.org/10.2147/IJWH.S29427> | Exclusion reason: Wrong age group; |
| Zakar 2016 | Domestic violence against rural women in Pakistan: An issue of health and human rights | Journal of Family Violence | 31 | 1 | 15-25 | <http://dx.doi.org/10.1007/s10896-015-9742-6> | Exclusion reason: Wrong age group; |
| Zakar 2013 | Spousal violence against women and its association with women's mental health in Pakistan | Health Care for Women International | 34 | 9 | 795-813 | <http://dx.doi.org/10.1080/07399332.2013.794462> | Exclusion reason: Wrong age group; |
| Zhang 2015 | Intimate partner violence among Hong Kong young adults: Prevalence, risk factors, and associated health problems | Journal of Interpersonal Violence | 30 | 13 | 2258-2277 | <http://dx.doi.org/10.1177/0886260514552442> | Exclusion reason: No differentiation between types of abuse |
| Zink 2005 | The Prevalence and Incidence of Intimate Partner Violence in Older Women in Primary Care Practices | Journal of General Internal Medicine | 20 | 10 | 884-888 | <http://dx.doi.org/10.1111/j.1525-1497.2005.0191.x> | Exclusion reason: No differentiation between types of abuse |

# Appendix C

Quality Assessment

*JBI* *Critical Appraisal Checklist for the 68 Included Studies*


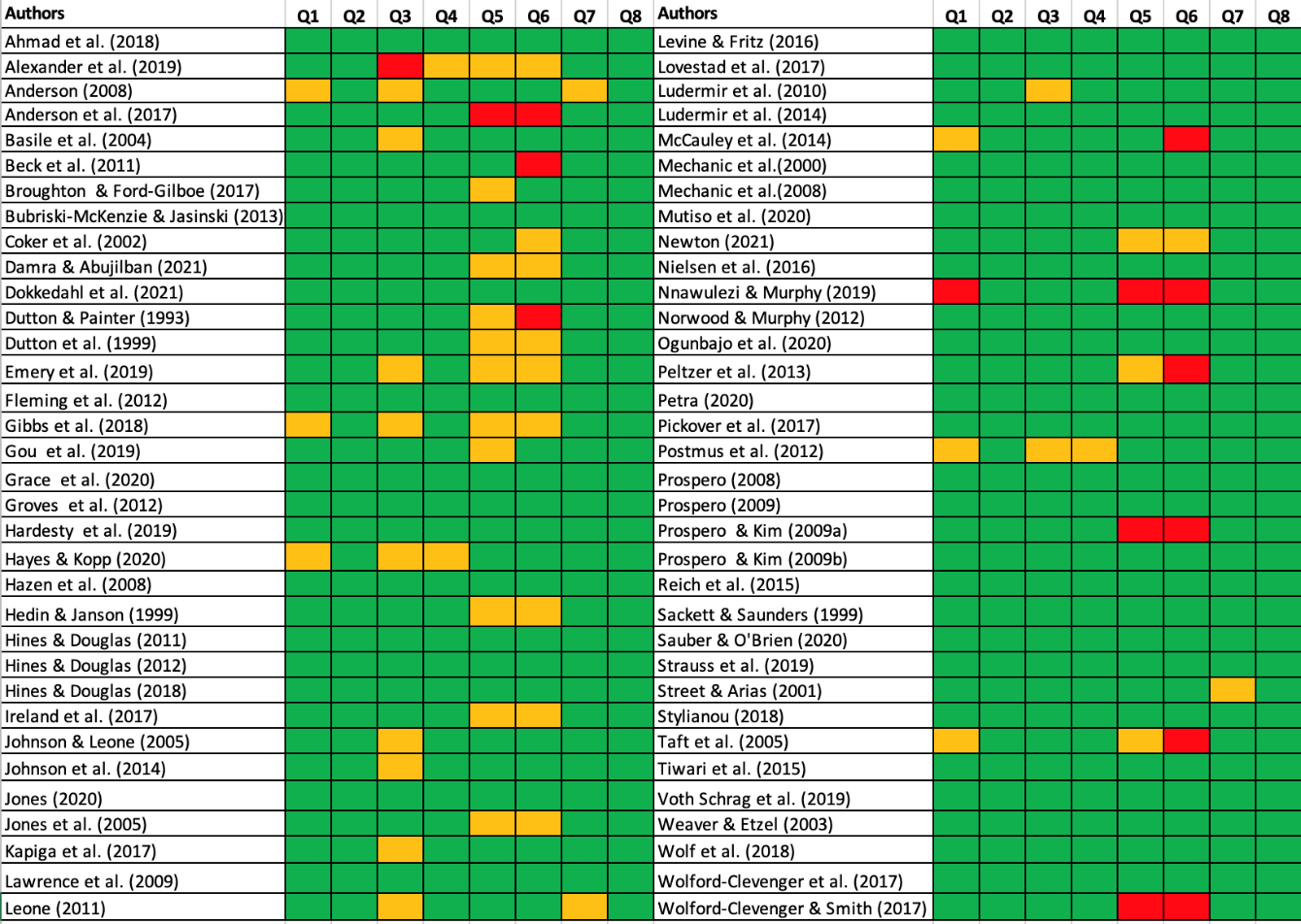


*Note*. Q1= “Were the criteria for inclusion in the sample clearly defined?”; Q2 = “Were the study subjects and the setting described in detail?”; (Q3 = “Was the exposure measured in a valid and reliable way?”; Q4 = “Were objective, standard criteria used for measurement of the condition?”; Q5 = “Were confounding factors identified?”; Q6 = “Were strategies to deal with confounding factors stated?”; Q7 = “Were the outcomes measured in a valid and reliable way?”; Q8 = “Was appropriate statistical analysis used?”. Yes = green, No = red, Unclear = orange.

# Appendix D

Measures References

Andresen, E.M., Malmgren, J.A., Carter, W.B., & Patrick, D.L. (1994). Screening for Depression in Well Older Adults: Evaluation of a Short Form of the CES-D. American. *Journal of Preventive Medicine*, *10*(2), 77-84. <https://doi.org/10.1016/S0749-3797(18)30622-6>

Bachman, J. G., Johnston, L. D., O’Malley, P. M., & Schulenberg, J. E. (2011). *The Monitoring the Future project after thirty-seven years: design and procedures*. Institute for Social Research, The University of Michigan. <http://www.monitoringthefuture.org/pubs/occpapers/mtf-occ76.pdf>

Beck, A. T., Epstein, N., Brown, G., & Steer, R. A. (1988). An inventory for measuring clinical anxiety: Psychometric properties. *Journal of Consulting and Clinical Psychology, 56*(6), 893–897. [https://doi.org/10.1037/0022-006X.56.6.893](https://psycnet.apa.org/doi/10.1037/0022-006X.56.6.893)

Beck, A. T., Steer, R. A., & Brown, G. K. (1996). *Manual for the Beck depression inventory-II*. San Antonio, TX: Psychological Corporation.

Beck, A.T., Ward, C. H., Mendelson, M., Mock, J., & Erbaugh, J. (1961) An inventory for measuring depression. *Archives of General Psychiatry,* *4*, 561-571

Beusenberg, M., Orley, John H., & World Health Organization. Division of Mental Health. (‎1994)‎. *A User's guide to the self reporting questionnaire (‎SRQ*/ compiled by M. Beusenberg and J. Orley. World Health Organization. <https://apps.who.int/iris/handle/10665/61113>

Blake, D., Weathers, F., Nagy, L., Kaloupek, D., Klauminzer, G., Charney, D., et al. (1990). *Clinician-administered PTSD scale (CAPS)*. Boston: National Center for Post-Traumatic Stress Disorder, Behavioral Science Division.

Blanchard, E. B., Jones-Alexander, J., Buckley, T. C., & Forneris, C. A. (1996). Psychometric properties of the PTSD Checklist (PCL). *Behaviour research and therapy*, *34*(8), 669–673. <https://doi.org/10.1016/0005-7967(96)00033-2>

Block, C. (2000). *Chicago Women’s Health Risk Study, 1995–1998* [Computer File ICPSR]. Chicago: Illinois Criminal Justice Information Authority. Borenstein, M., Hedges, L. V., Higgins, J. P., & Rothstein, H. R. (2014). *Comprehensive meta-analysis* (Version 3.3.070) [Computer software]. Biostat. <https://www.meta-analysis.com/>

Briere, J., & Runtz, M. (1989). The Trauma Symptom Checklist (TSC-33). *Journal of Interpersonal Violence*, *4*(2), 151–163. <https://doi.org/10.1177/088626089004002002>

Brown, J. B., Lent, B., Brett, P. J., Sas, G., & Pederson, L. L. (1996). Development of the Woman Abuse Screening Tool for use in family practice. *Family Medicine*, *28*(6), 422-428.

Brown, T. A., Barlow, D. H., & DiNardo, P. A. (1994). *Anxiety disorders interview schedule adult version: Client interview schedule*. Graywind Publications Incorporated.

Cloitre, M., Shevlin, M., Brewin, C. R., Bisson, J. I., Roberts, N. P., Maercker, A., Karatzias, T., & Hyland, P. (2018). The International Trauma Questionnaire: development of a self-report measure of ICD-11 PTSD and complex PTSD. *Acta Psychiatrica Scandinavica, 138*(6), 536-546. <https://doi.org/10.1111/acps.12956>

Coleman, F. L. (1997). Stalking Behavior and the Cycle of Domestic Violence. *Journal of Interpersonal Violence*, *12*(3), 420–432. <https://doi.org/10.1177/088626097012003007>

Cox, J. L., Holden, J. M., & Sagovsky, R. (1987). Detection of postnatal depression: development of the 10-item Edinburgh Postnatal Depression Scale. *The British Journal of Psychiatry*, *150*(6), 782-786

Derogatis, L. R. (1983). SCL-90-R: *Administration, scoring, and procedures manual—II*. Baltimore: Clinical Psychometric Research.

Derogatis, L. R. (1993*). Brief Symptom Inventory: Administration, scoring, and procedures manual*. Minneapolis, MN: NCS Pearson, Inc.

Dutton, M. A., Goodman, L. A., & Schmidt, R. J. (2005). *Development and validation of a coercive control measure for intimate partner violence: Final technical report* (US DOJ Document No. 214438). U.S. Department of Justice.

Dutton, M. A., Goodman, L. A., Terrell, D., Schmidt, R. J., & Fujimoto, A. (2007). Coercion in intimate partner relationships (CPR): A measure of demands, surveillance, coercive tactics, and behavioral response to coercive tactics. *Final report submitted to National Institute of Justice*.

Eaton, W. W., Smith, C., Ybarra, M., Muntaner, C., & Tien, A. (2004). Center for Epidemiologic Studies Depression Scale: Review and Revision (CESD and CESD-R). In M. E. Maruish (Ed.), *The use of psychological testing for treatment planning and outcomes assessment: Instruments for adults* (pp. 363–377). Lawrence Erlbaum Associates Publishers.

Ewing, J. (1984). Detecting alcoholism: The CAGE Questionnaire. *Journal of the American Medical Association*, *252*, 1905-1907. <https://doi:10.1001/jama.1984.03350140051025>

Foa, E., Cashman, L., Jaycox, L., & Perry, K. (1997). The validation of a self-report measure of PTSD: The Post-traumatic Diagnostic Scale (PDS). *Psychological Assessment, 9*, 445–451.

Foa, E. B., Riggs, D. S., Dancu, C. V., & Rothbaum, B. O. (1993a). *PTSD Symptom Scale-Interview Version (PSS-I)* [Database record]. APA PsycTests. [https://doi.org/10.1037/t05176-000](https://psycnet.apa.org/doi/10.1037/t05176-000)

Foa, E. B., Riggs, D. S., Dancu, C. V., & Rothbaum, B. O. (1993b). Reliability and validity of a brief instrument for assessing post‐traumatic stress disorder. *Journal of traumatic stress*, *6*(4), 459-473.

Ford-Gilboe, M., Wuest, J., Varcoe, C., Davies, L., Merritt-Gray, M., Campbell, J., & Wilk, P. (2009). Modelling the effects of intimate partner violence and access to resources on women’s health in the early years after leaving an abusive partner. *Social Science & Medicine*, *68*(6), 1021–1029. <https://doi.org/10.1016/j.socscimed.2009.01.003>

Graham-Kevan, N., & Archer, J. (2003a). Intimate terrorism and common couple violence. A test of Johnson's predictions in four British samples. *Journal of Interpersonal Violence, 18*(11), 1247-1270. <https://doi.org/10.1177/0886260503256656>

Graham-Kevan, N., & Archer, J. (2003b). Physical Aggression and Control in Heterosexual Relationships: The Effect of Sampling. *Violence and victims, 18*(2), 181-196. <https://doi.org/10.1891/vivi.2003.18.2.181>

Gray, M. J., Litz, B. T., Hsu, J. L., & Lombardo, T. W. (2004). Psychometric Properties of the Life Events Checklist. *Assessment*, *11*(4), 330–341. <https://doi.org/10.1177/1073191104269954>

Hays, R., Sherbourne, C., & Mazel, R. (1995). *User’s manual for the medical outcomes study (MOS) core measures of health related quality of life* (Publication No. MR-162-RC). RAND.

Hegarty, K., Bush, R., Sheehan, M. (2005). Composite Abuse Scale: Further development and assessment of reliability and validity of a multidimensional partner abuse measure in clinical settings. *Violence Victims, 20*(5), 529-547. <https://doi.org/10.1891/vivi.2005.20.5.529>

Hegarty, K., Sheehan, M., Schonfeld, C. (1999). A multidimensional definition of partner abuse: Development and preliminary validation of the Composite Abuse Scale. *Journal of Family Violence, 14*(4), 399-415.

Kellner, R. (1987). A symptom questionnaire. *Journal of Clinical Psychiatry*, *48*(7), 268-274.

Kessler, R. C., Andrews, G., Mroczek, D., Ustun, B., & Wittchen, H.-U. (1998). The World Health Organization Composite International Diagnostic Interview short-form (CIDI-SF). *International Journal of Methods in Psychiatric Research*, *7*(4), 171–185. <https://doi.org/10.1002/mpr.47>

Kilpatrick, D. G., Acierno, R., Resnick, H. S., Saunders, B. E., & Best, C. L. (1997). A 2-year longitudinal analysis of the relationships between violent assault and substance use in women. *Journal of Consulting and Clinical Psychology, 65*(5), 834–847. [https://doi.org/10.1037/0022-006X.65.5.834](https://psycnet.apa.org/doi/10.1037/0022-006X.65.5.834)

Krog, T., & Duel, M. (2003). Traume symptom checkliste (TSC): En validering og revidering. *Psykologisk Studieskriftserie*, *6*(4), 1–163.

Lang, A. J., & Stein, M. B. (2005). An abbreviated PTSD checklist for use as a screening instrument in primary care. *Behaviour research and therapy*, *43*(5), 585–594. <https://doi.org/10.1016/j.brat.2004.04.005>

Leone, J. M., Johnson, M. P., & Cohan, C. L. (2007). Victim Help Seeking: Differences Between Intimate Terrorism and Situational Couple Violence. *Family Relations*, *56*(5), 427–439. <https://doi.org/10.1111/j.1741-3729.2007.00471.x>

Linehan, M. M., Comtois, K. A., Brown, M. Z., Heard, H. L., & Wagner, A. (2006). Suicide Attempt Self-Injury Interview (SASII): development, reliability, and validity of a scale to assess suicide attempts and intentional self-injury. *Psychological assessment*, *18*(3), 303–312. <https://doi.org/10.1037/1040-3590.18.3.303>

Lovibond, S. H., & Lovibond, P.F. (1995). *Manual for the Depression Anxiety Stress Scales* (2nd. Ed.). Sydney: Psychology Foundation.

Marshall, L. L. (1992). Development of the Severity of Violence Against Women Scales *Journal of Family Violence, 7*, 103–121 <https://doi.org/https://doi.org/10.1007/BF00978700>

Mollica, R. F., Caspi-Yavin, Y., Bollini, P., Truong, T., Tor, S., & Lavelle, J. (1992). The Harvard Trauma Questionnaire: Validating a cross-cultural instrument for measuring torture, trauma, and posttraumatic stress disorder in Indochinese refugees. *Journal of Nervous and Mental Disease, 180*(2), 111–116. [https://doi.org/10.1097/00005053-199202000-00008](https://psycnet.apa.org/doi/10.1097/00005053-199202000-00008)

Parloff, M. B., Kelman, H. C., & Frank, J. D. (1954). Comfort, effectiveness, and self-awareness as criteria of improvement in psychotherapy. *American Journal of Psychiatry*, *111*(5), 343-352. <https://doi.org/10.1176/ajp.111.5.343>

Pokman, V., Rossi, F. S., Holtzworth-Munroe, A., Applegate, A. G., Beck, C. J. A., & D’Onofrio, B. M.. (2014). Mediator’s Assessment of Safety Issues and Concerns (MASIC). *Assessment*, *21*(5), 529–542. <https://doi.org/10.1177/1073191114528372>

Postmus, J. L., Plummer, S., & Stylianou, A. M. (2016a). Measuring economic abuse in the lives of survivors: Revising the Scale of Economic Abuse. *Violence Against Women*, *2*, 692-703. [https://doi.org/10.1177/1077801215610012](https://doi.org/10.1177%2F1077801215610012)

Postmus, J. L., Stylianou, A. M., & McMahon, S. (2016b). The Abusive Behavior Inventory–Revised. Journal of Interpersonal Violence, 31(7), 2867–2888. <https://doi.org/10.1177/0886260515581882>

Prins, A., Ouimette, P., Kimerling, R., Cameron, R. P., Hugelshofer, D. S., Shaw-Hegwer, J., Thrailkill, A., Gusman, F.D., Sheikh, J. I. (2003). The Primary Care PTSD Screen (PCPTSD): Development and operating characteristics (PDF). *Primary Care Psychiatry, 9,* 9-14. <https://doi.org/10.1185/135525703125002360>

Pulerwitz, J., Amaro, H., De Jong, W., Gortmaker, S. L., & Rudd, R. (2002). Relationship power, condom use and HIV risk among women in the USA. *AIDS Care, 14*, 789-800. <https://doi:10.1080/0954012021000031868>

Radloff, L. S. (1977). The CES-D scale: A self report depression scale for research in the general population. *Applied Psychological Measurements,1*, 385-401.

Robins, L. N., Helzer, J. E., Ratcliff, K. S., & Seyfried, W. (1982). Validity of the Diagnostic Interview Schedule, Version II: DSM–III diagnoses. *Psychological Medicine, 12*, 855–870.

Saunders, D. G. (1994). Posttraumatic Stress Symptom Profiles of Battered Women: A Comparison of Survivors in Two Settings. *Violence and Victims*, *9*(1), 31–44. <https://doi.org/10.1891/0886-6708.9.1.31>

Saunders, J. B., Aasland, O. G., Babor, T. F., De La Fuente, J. R., & Grant, M. (1993). Development of the Alcohol Use Disorders Identification Test (AUDIT): WHO Collaborative Project on Early Detection of Persons with Harmful Alcohol Consumption-II. *Addiction*, *88*(6), 791–804. <https://doi.org/10.1111/j.1360-0443.1993.tb02093.x>

Sheehan, D. V., Lecrubier, Y, Sheehan, KH, Janavs, J, Weiller, E, Keskiner, A, et al. (1997). The validity of the Mini International Neuropsychiatric Interview (MINI) according to the SCID-P and its reliability. *European Psychiatry, 12*(5), 232–241.

Shepard, M. F., & Campbell, J. A. (1992). The Abusive Behavior Inventory. *Journal of Interpersonal Violence*, *7*(3), 291–305. <https://doi.org/10.1177/088626092007003001>

Sheridan, D. (1998). *Measuring harassment of battered women* [Doctoral Dissertation, Oregon Health Sciences University]. OHSU Library WY4 S552 1998.

Smith, P. H., Smith, J. B., & Earp, J. A. L. (1999). Beyond the measurement trap: a reconstructed conceptualization and measurement of woman battering. *Psychology of Women Quarterly* (1), 177.

Smith, S. G., Chen, J., Basile, K. C., Gilbert, L. K., Merrick, M. T., Patel, N., Walling, M., & Jain, A. (2017). *The National Intimate Partner and Sexual Violence Survey (NISVS): 2010–2012 state report*. Atlanta, GA: National Center for Injury Prevention and Control, Centers for Disease Control and Prevention.

Spielberger, C. D., Gorsuch, R. L., Lushene, R., Vagg, P. R., & Jacobs, G. A. (1983). *Manual for the State-Trait Anxiety Inventory*. Palo Alto, CA: Consulting Psychologists Press

Spitzer, R. L., Kroenke, K., Williams, J. B. W., & Löwe, B. (2006). A Brief Measure for Assessing Generalized Anxiety Disorder. *Archives of Internal Medicine*, *166*(10), 1092. <https://doi.org/10.1001/archinte.166.10.1092>

Stephenson, R., & Finneran, C. (2013). The IPV-GBM Scale: A New Scale to Measure Intimate Partner Violence among Gay and Bisexual Men. *PLOS ONE*, *8*(6), e62592. <https://doi.org/10.1371/journal.pone.0062592>

Straus, M. A. (1990). The Conflict Tactics Scales and its critics: An evaluation and new data on validity and reliability. In MA Straus & RJ Gelles (Eds.), *Physical violence in American families: Risk factors and adaptations to violence in 8,145 families*. 49-73. Transaction Publishing.

Straus, M. A., Hamby, S. L., Boney-McCoy, S., & Sugarman, D. B. (1996). The Revised Conflict Tactics Scale (CTS2). *Journal of Family Issues, 17*(3), 283-316 <https://doi.org/https://doi-org.ezp.lib.unimelb.edu.au/10.1177/019251396017003001>

Stuart, G. L., Moore, T. M., Kahler, C. W., & Ramsey, S. E. (2003). Substance abuse and relationship violence among men court‐referred to batterers’ intervention programs. *Substance Abuse*, *24*(2), 107–122. <https://doi.org/10.1080/08897070309511539>

Tjaden, P., & Thoennes, N. (1999). *Extent, nature, and consequences of intimate partner vio- lence: Findings from the National Violence Against Women Survey*. National Institute of Justice/Centers for Disease Control and Prevention.

Tiwari, A., Fong, D. Y. T., Chan, K. L., Leung, W. C., Parker, B., & Ho, P. C. (2007). Identifying intimate partner violence: comparing the Chinese abuse assessment screen with the Chinese revised conflict tactics scales. *BJOG: An International Journal of Obstetrics & Gynaecology*, *114*(9), 1065-1071.

Tolman, R. M. (1989). The development of a measure of psychological maltreatment of women by their male partners. *Violence and victims, 4*(3), 159-177. <https://doi.org/10.1891/0886-6708.4.3.159>

Tolman, R. M. (1999). The Validation of the Psychological Maltreatment of Women Inventory. *Violence and Victims 14*(1), 25-37. <https://doi.org/10.1891/0886-6708.14.1.25>

Vreven, D. L., Gudanowski, D. M., King, L. A., & King, D. W. (1995). The civilian version of the Mississippi PTSD scale: A psychometric evaluation. *Journal of Traumatic Stress*, *8*(1), 91–109. <https://doi.org/10.1002/jts.2490080107>

Ware, J. E. , Jr., & Sherbourne, C. D. (1992). The MOS 36-item short-form health survey (SF-36). I. Conceptual framework and item selection. *Medical Care* *30*(6), 473-83.

Weathers, F. W., Blake, D. D., Schnurr, P.P., Kaloupek, D.G., Marx, B.P., & Keane, T.M. (2013a). *The Life Events Checklist for DSM-5 (LEC-5*). <https://www.ptsd.va.gov/>

Weathers, F. W., Litz, B. T., Herman, D. S., Huska, J. A., & Keane, T. M. (1993). *The PTSD Checklist (PCL): Reliability, validity, and diagnostic utility*. Paper presented at the International Society for Traumatic Stress Studies, San Antonio, TX.

Weathers, F. W., Litz, B.T., Keane, T.M., Palmieri, P.A., Marx, B.P., & Schnurr, P.P. (2013b). *The PTSD Checklist for DSM-5 (PCL-5)*. National Center for PTSD. <https://www.ptsd.va.gov/>

Weiss, D. S., & Marmar, C. R. (1997). The Impact of Event Scale—Revised. In J. P. Wilson & T. M. Keane (Eds.), *Assessing psychological trauma and PTSD* (pp. 399–411). The Guilford Press.

Winokur, A., Winokur, D. F., Rickels, K., & Cox, D. S. (1984). Symptoms of emotional distress in a family planning service: stability over a four-week period. *The British journal of psychiatry : the journal of mental science*, *144*, 395–399. <https://doi.org/10.1192/bjp.144.4.395>

Zigmond, A. S., & Snaith, R. P. (1983). The hospital anxiety and depression scale. *Acta psychiatrica scandinavica*, *67*(6), 361-370.

Zimmerman, M., & Mattia, J. I. (2001). The Psychiatric Diagnostic Screening Questionnaire: development, reliability and validity. *Comprehensive psychiatry*, *42*(3), 175–189. <https://doi.org/10.1053/comp.2001.23126>

# Appendix E

Forest Plots

**Figure E1**

*Forest Plot for Coercive Control and PTSD*


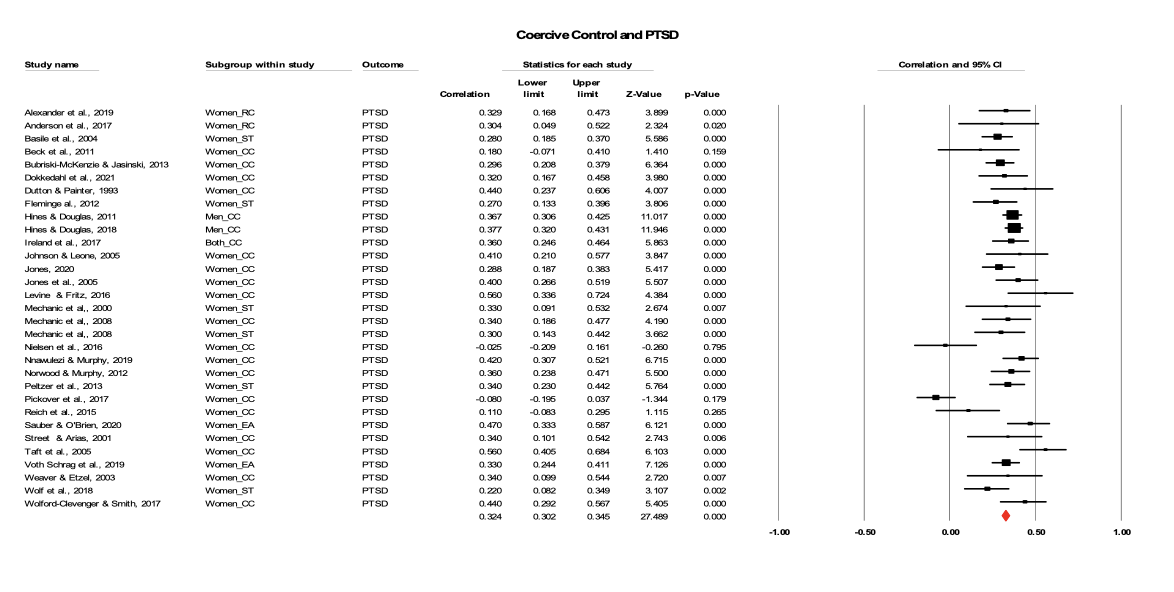


**Figure E2**

*Forest Plot for Coercive Control and Depression*


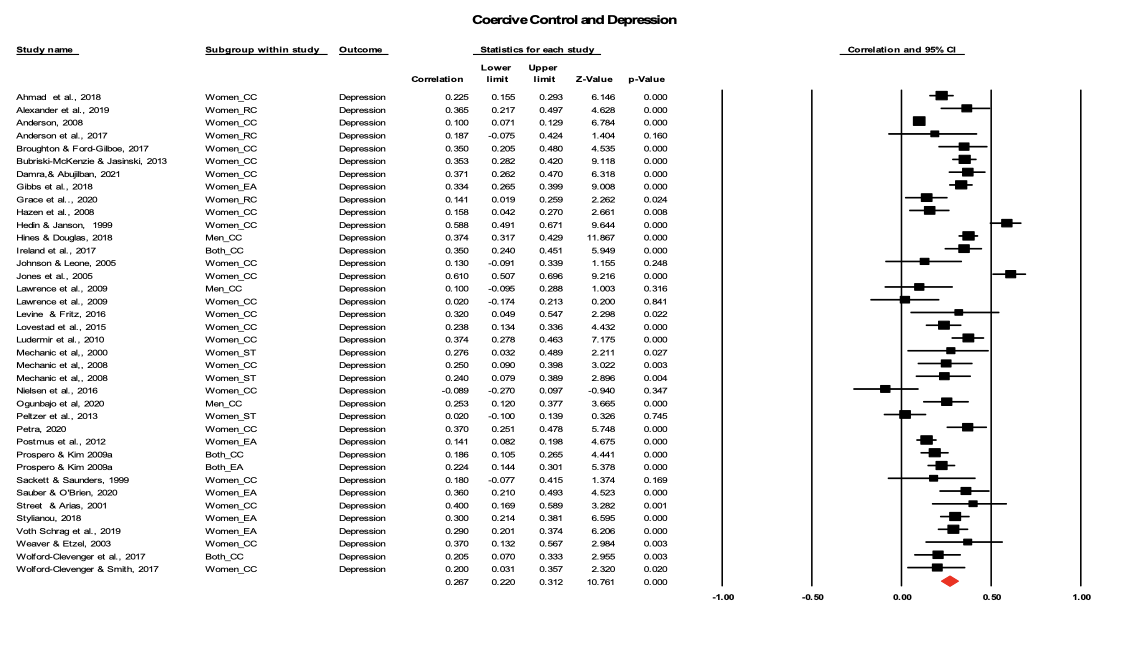


# Appendix F

Subgroup Analyses

**Table F1**

*Subgroup Analyses for Coercive Control, PTSD and Depression*

| Subgroup | Studies *k* | Effect Sizes *k* | | | | *r* | | 95% CI | *I^2^ %* |
| --- | --- | --- | --- | --- | --- | --- | --- | --- | --- |
|  |  | Total | Women | Men | Both | Range | Mean |  |  |
| PTSD |  |  |  |  |  |  |  |  |  |
| Types of IPV |  |  |  |  |  |  |  |  |  |
| CC | 21 | 21 | 18 | 2 | 1 | -.08 - .56 | .33* | [.26, .39] | 79.51* |
| EA, ST, RC | 10 | 10 | 10 | - | - | .27 - .35 | .32* | [.27,.35] | 0.00 |
| Study Setting |  |  |  |  |  |  |  |  |  |
| DV Support/Shelter | 10 | 10 | 10 | - | - | .32 -.56 | .40* | [.35,.45] | 7.28 |
| Community^1^ | 11 | 11 | 9 | 2 | 1 | -.08 -.41 | .26* | [.16,.35] | 86.85* |
|  |  |  |  |  |  |  |  |  |  |
| Depression |  |  |  |  |  |  |  |  |  |
| Types of IPV |  |  |  |  |  |  |  |  |  |
| CC | 26 | 27 | 21 | 3 | 3 | -.09 - .59 | .28* | [.26, .39] | 89.82* |
| EA, ST, RC | 12 | 12 | 11 | - | 1 | .18 - .30 | .24* | [.18, .30] | 74.48* |
| Study Setting |  |  |  |  |  |  |  |  |  |
| DV Support/Shelter | 6 | 6 | 6 | - | - | .18 - .40 | .27* | [.19, .35] | 0 |
| Community^1^ | 20 | 21 | 16 | 2 | 3 | -.09 - .59 | .28* | [.21, .35] | 91.76* |

*Note*. *CC* = Coercive Control (excluding economic abuse, stalking, reproductive coercion) ; *EA* = economic abuse; *ST* = stalking; *RC* = reproductive coercion; *PMWI-DI* = Psychological Maltreatment of Women Inventory dominance/isolation subscale; *PCL-C* = PTSD Checklist-Civilian (Weathers et al., 1993); *PCL-C-IPV =* PTSD Checklist-Civilian Modified to IPV experience (Weathers et al., 1993); *BDI* = Beck Depression Inventory (Beck et al., 1961); *BDI-II* = Beck Depression Inventory (Beck et al., 1996); ^1^community refers to all study settings aside from DV Support/Shelter; **P* = < .001 ** *P* = < .05

# Appendix G

Risk of Publication Bias

**Table G1**

*Risk of Publication Bias for all Meta-analyses*

|  | Effect Sizes | Trim and Fill | Classic  Fail-Safe *N* | Orwin’s  Fail-Safe *N* |
| --- | --- | --- | --- | --- |
| Correlation between | *k* | Imputed Studies | *N* | *r* ≤ .10 |
| ALL CC – PTSD | 31 | 9 | 5123 | 73 |
| CC – PTSD | 21 | 5 | 2410 | 51 |
| EA, ST, RC – PTSD | 10 | 0 | 496 | 23 |
| PSY – PTSD | 19 | 0 | 2055 | 47 |
| ALL CC - Depression | 39 | 0 | 7702 | 49 |
| CC – Depression | 27 | 0 | 3868 | 34 |
| EA, ST, RC – Depression | 12 | 1 | 642 | 16 |
| PSY – Depression | 19 | 0 | 2422 | 44 |
| *Note*. CC = Coercive Control; ALL CC = CC including economic abuse (EA); stalking (ST); reproductive coercion (RC); PSY = Psychological IPV | | | | |
